# Supplementary material for: Genetic architecture of sleep in a genome wide association study of device measured sleep traits
Source: Nat Commun. 2026 Apr 1;17:4715. doi: 10.1038/s41467-026-71252-y (PMC13212655; doi:10.1038/s41467-026-71252-y)

Supplementary Figure 1. Participant flow diagram for the GWAS of wearable device-derived sleep measurements in the UK Biobank.

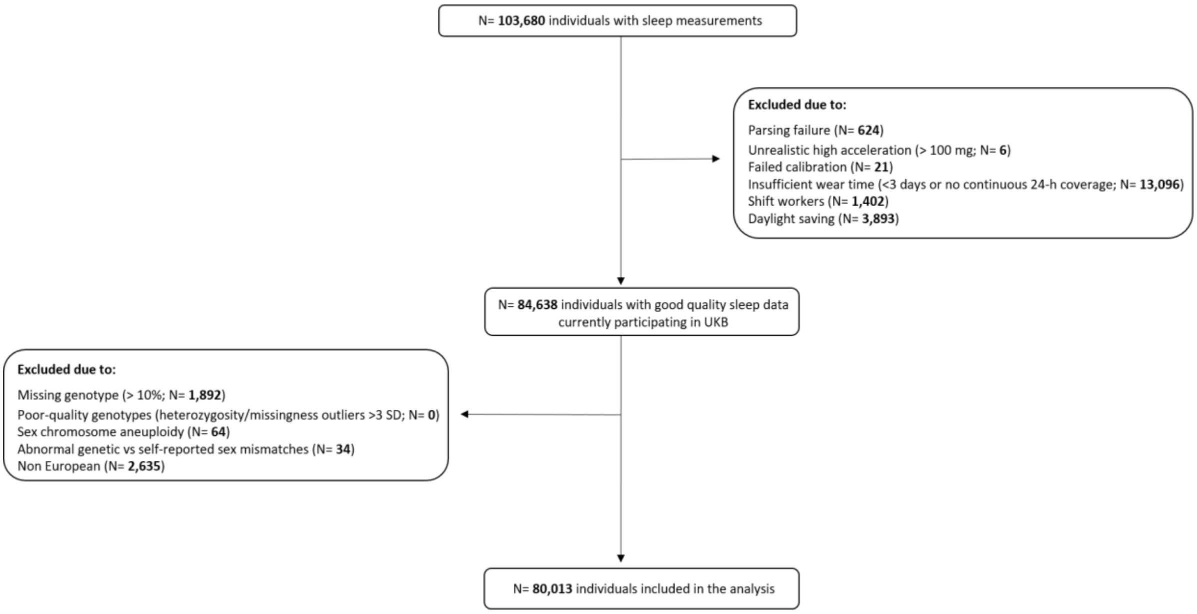

Supplementary Figure 2. Q-Q plots and LD Score regression estimates (SNP-based heritability (h2SNP), genomic control lambda (λ), intercept, and ratio) for genome-wide association analysis of sleep traits: a) night-time sleep duration, b) sleep efficiency, c) REM sleep duration, and d) Non-REM sleep duration.

a)

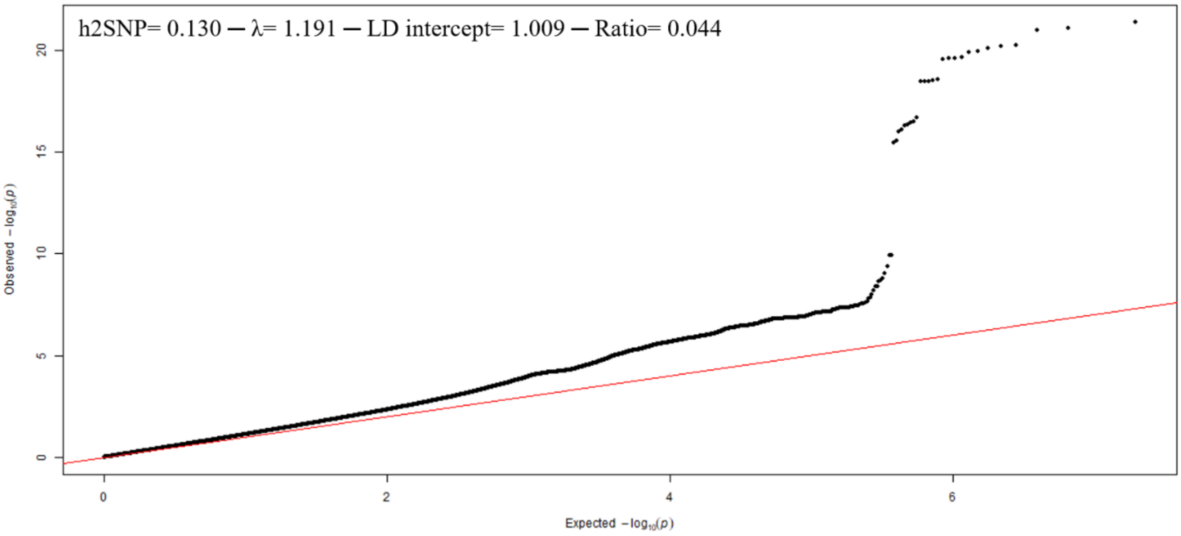

b)

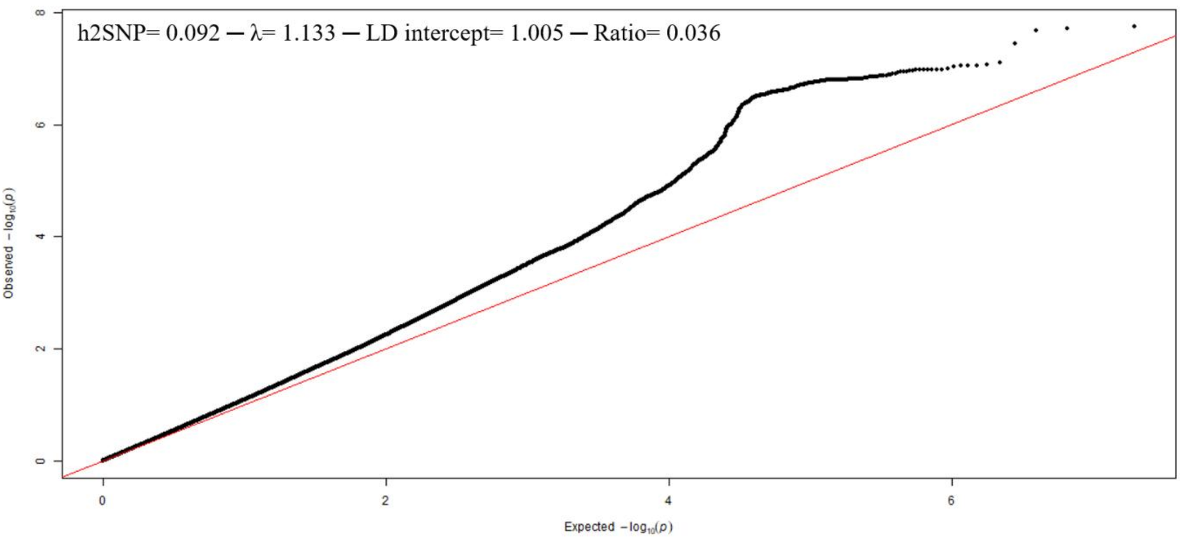
h2SNP= 0.092 — λ= 1.133 — LD intercept= 1.005 — Ratio= 0.036
Observed −log10(p)
Expected −log10(p)
0
2
4
6
8

c)

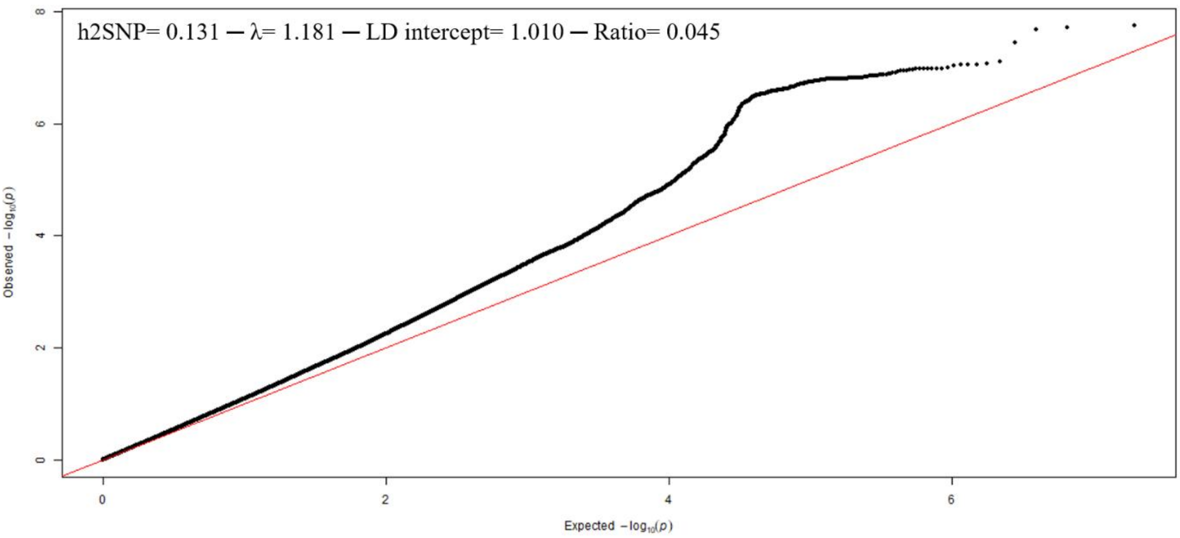
h2SNP= 0.131 — λ= 1.181 — LD intercept= 1.010 — Ratio= 0.045
Observed −log10(p)
Expected −log10(p)
0
2
4
6
8

d)

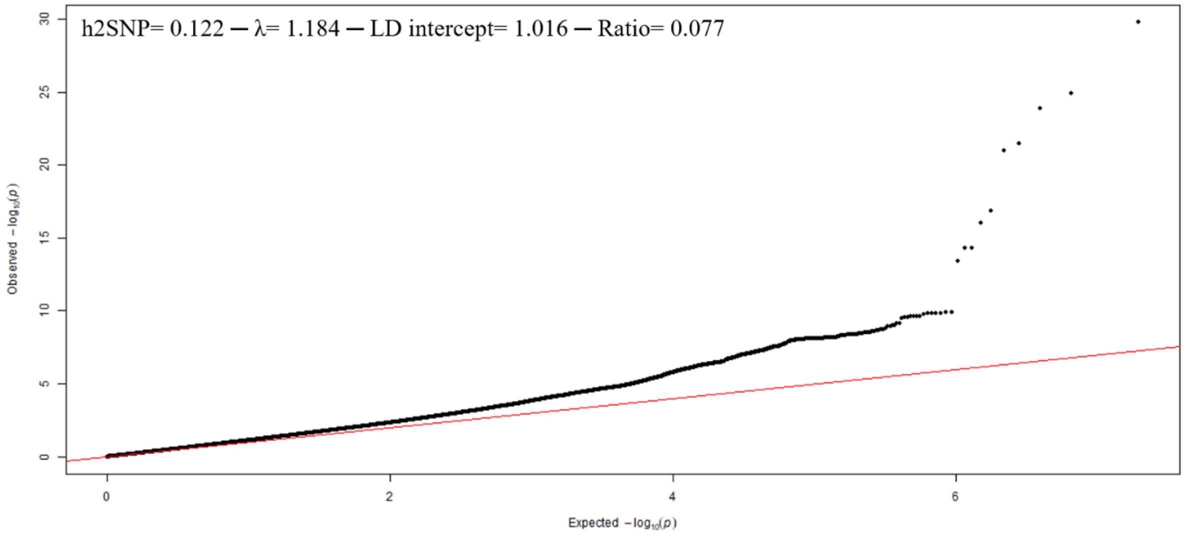

Supplementary Figure 3. Phenotypic (Pearson correlation - r) and genetic (measured by LDSC - r_g) correlation between sleep traits.

	Night-time sleep	Sleep efficiency	REM sleep	Non-REM sleep
**Sleep efficiency**	$r= 0.42; p< 2.2^{-16}$<br>$r_g= 0.45; p< 2.2^{-16}$			
**REM sleep**	$r= 0.36; p< 2.2^{-16}$<br>$r_g= 0.43; p< 2.2^{-16}$	$r= 0.18; p< 2.2^{-16}$<br>$r_g= 0.22; p= 9.53^{-6}$		
**Non-REM sleep**	$r= 0.79 ; p< 2.2^{-16}$<br>$r_g= 0.79; p< 2.2^{-16}$	$r= 0.31; p< 2.2^{-16}$<br>$r_g= 0.35 ; p= 1.90^{-12}$	$r= -0.29; p< 2.2^{-16}$<br>$r_g= -0.21; p= 1.74^{-5}$	

Supplementary Figure 4. Phenotypic associations among accelerometer-derived measures of night-time sleep, REM, and NREM duration.

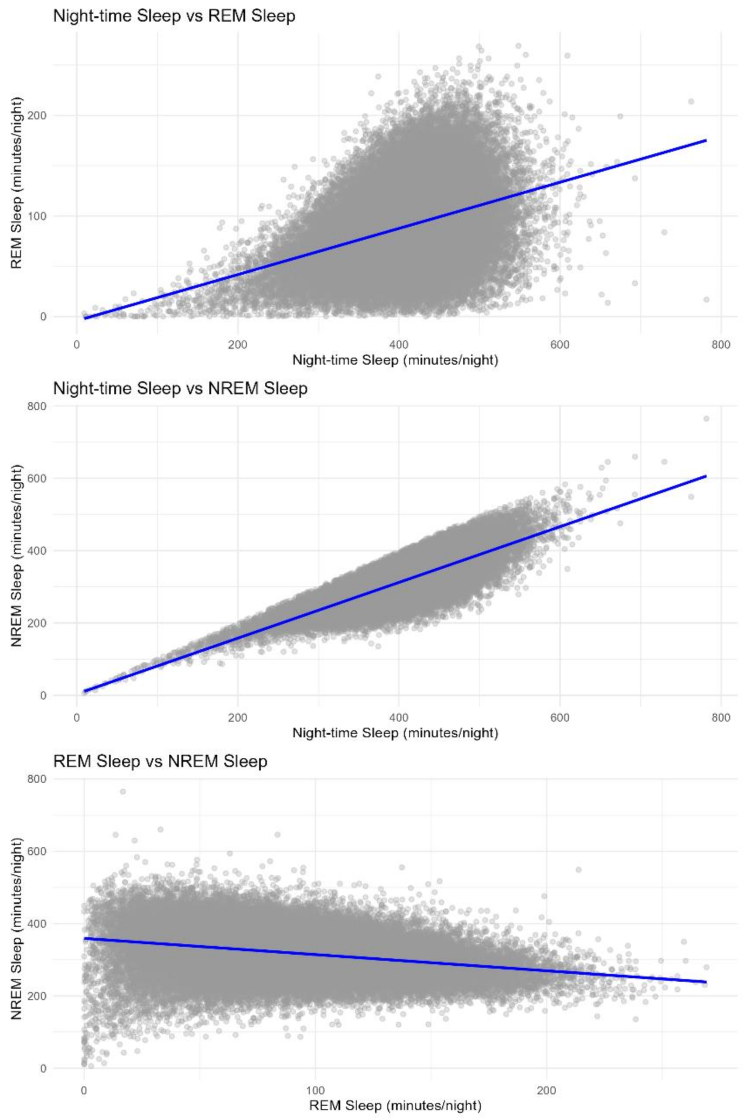

Supplementary Figure 5. Manhattan plot of sleep traits: a) night-time sleep duration, b) sleep efficiency, c) REM sleep duration, and d) Non-REM sleep duration.

a)

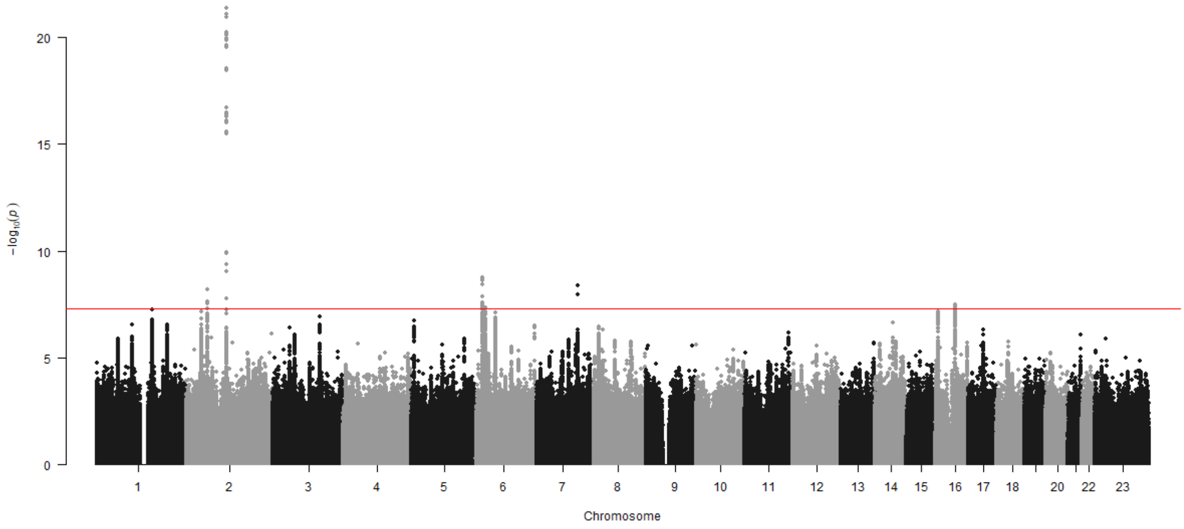

b)

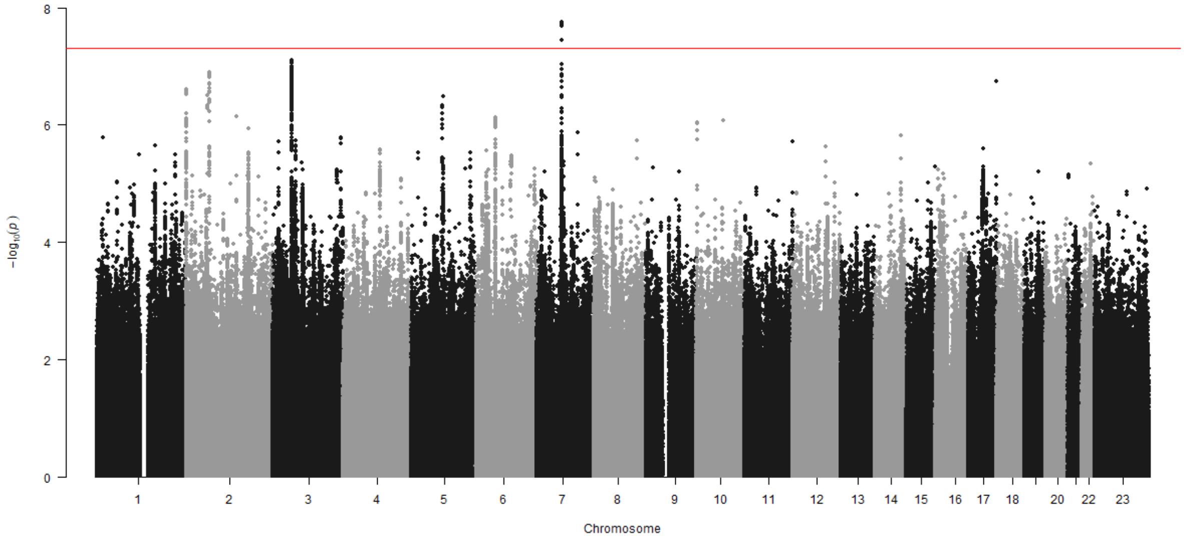

c)

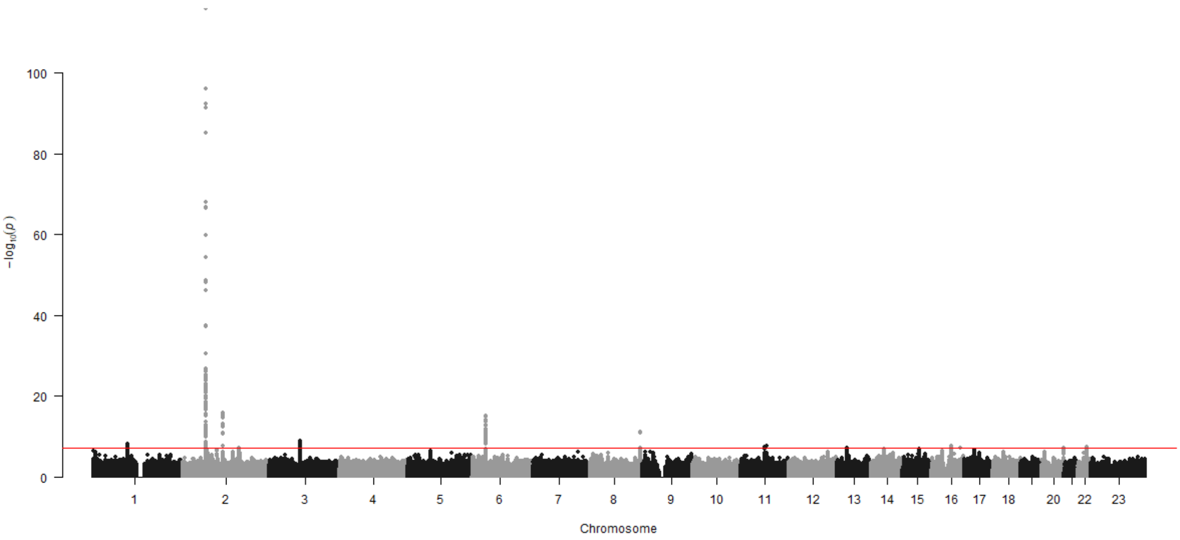
100
80
60
40
20
0
$-\log_{10}(p)$
1
2
3
4
5
6
7
8
9
10
11
12
13
14
15
16
17
18
20
22
23
Chromosome

d)

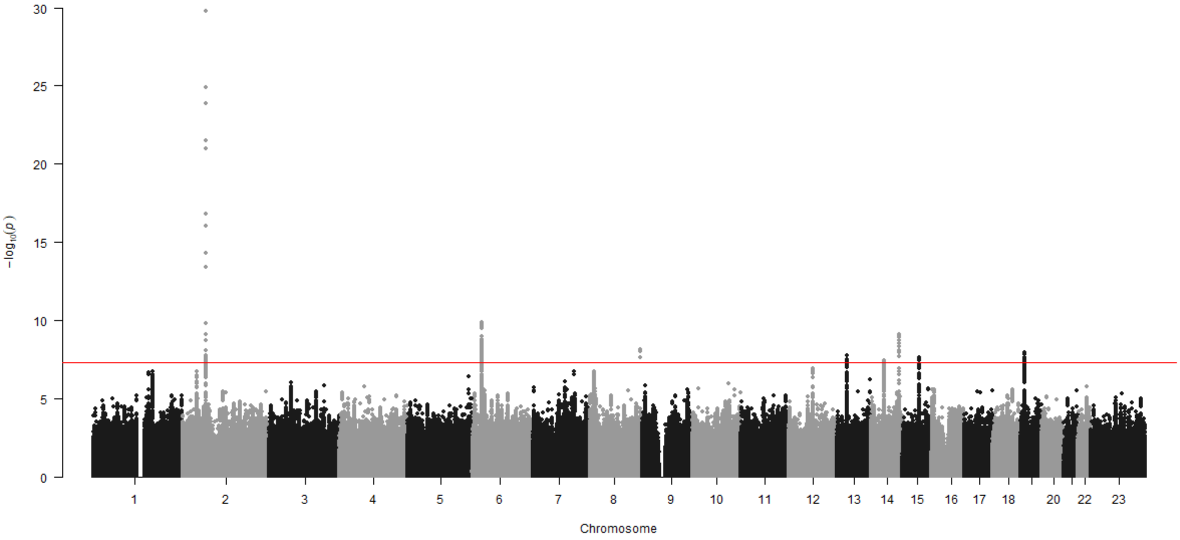
30
25
20
15
10
5
0
$-\log_{10}(p)$
1
2
3
4
5
6
7
8
9
10
11
12
13
14
15
16
17
18
20
22
23
Chromosome

Supplementary Figure 6. Locus zoom plots for associated loci across sleep traits (night-time sleep: i) 2p16.1, ii) 2q14.1, iii) 6p22.3, iv) 6p22.2, v) 7q31.1, and vi) 16q12.2; sleep efficiency: vii) 7q11.22; REM sleep: viii) 1p21.3, ix) 2p14, x) 2q14.1, xi) 3p11.1, xii) 6p21.2, xiii) 8q24.3, xiv) 11q13.2, xv) 11q13.4, xvi) 16q12.2, and xvii) 22q13.1; Non-REM sleep: xviii) 2p14, xix) 6p22.2, xx) 8q24.3, xxi) 13q14.2, xxii) 14q22.3, xxiii) 14q32.2, xxiv) 15q23, and xxv) 19p13.2). For loci 2q14.1 and 11q13.2, a proxy SNP in perfect LD was used for plotting due to the unavailability of the lead SNP.

i)

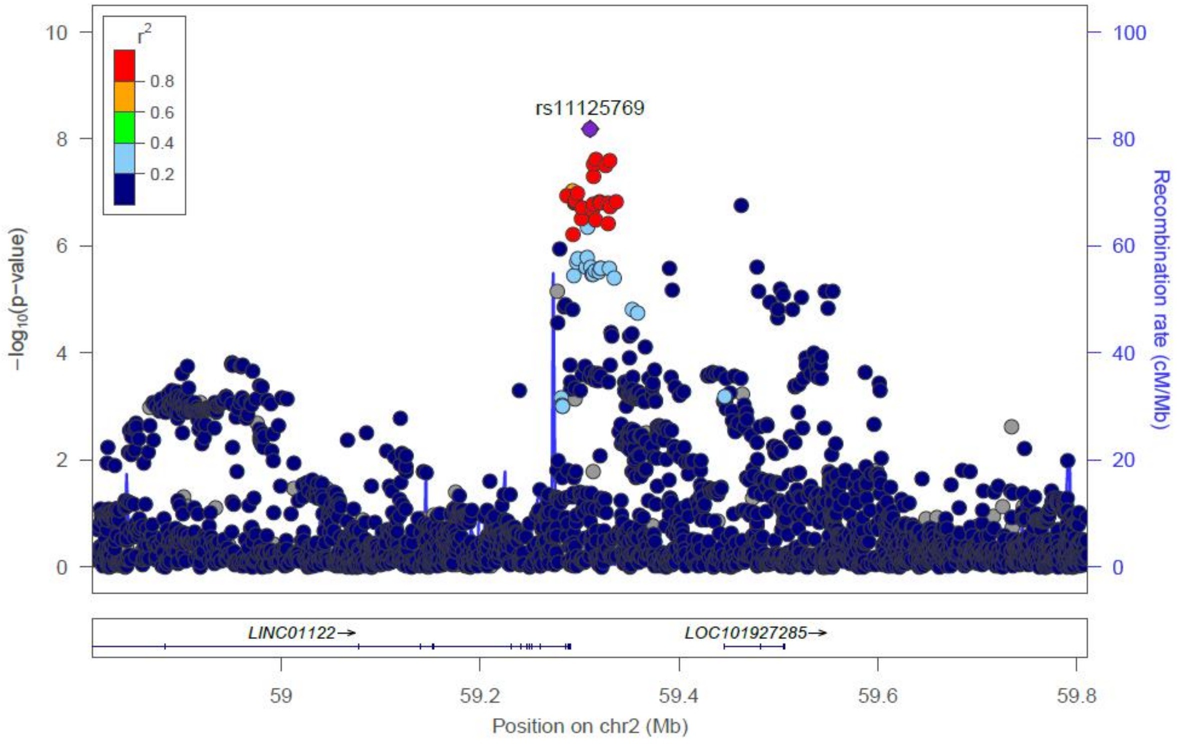

ii)

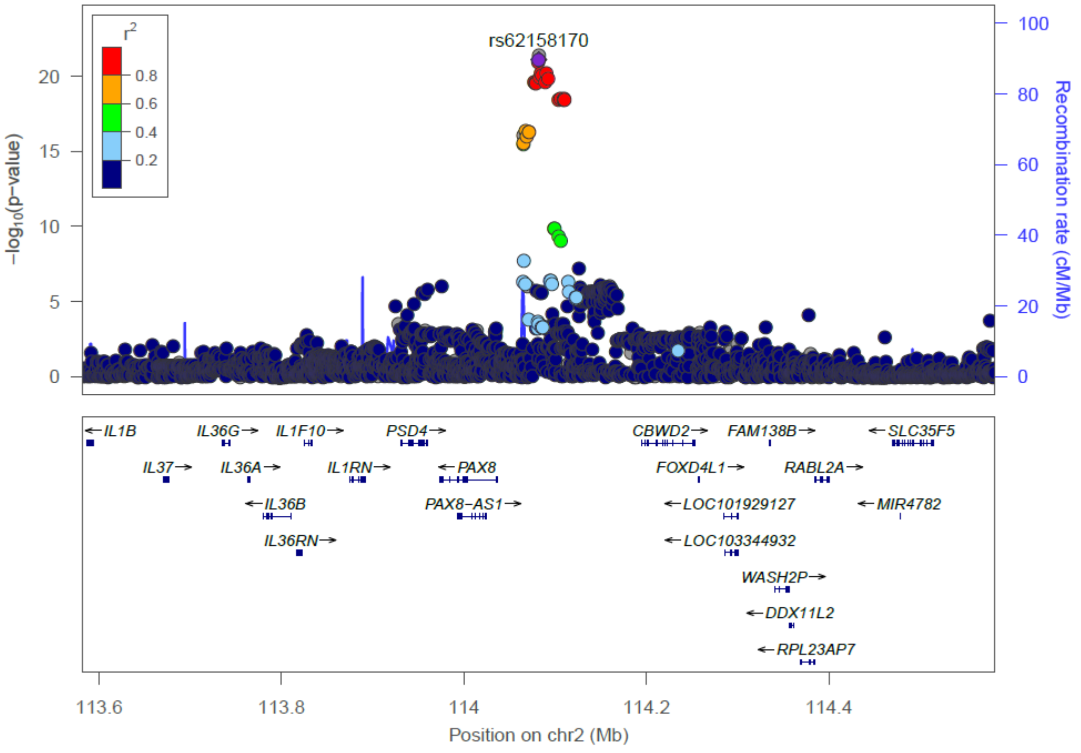
r2
0.8
0.6
0.4
0.2
rs62158170
−log10(p−value)
Recombination rate (cM/Mb)
IL1B
IL36G
IL1F10
PSD4
CBWD2
FAM138B
SLC35F5
IL37
IL36A
IL1RN
PAX8
FOXD4L1
RABL2A
IL36B
PAX8−AS1
LOC101929127
MIR4782
IL36RN
LOC103344932
WASH2P
DDX11L2
RPL23AP7
113.6
113.8
114
114.2
114.4
Position on chr2 (Mb)

iii)

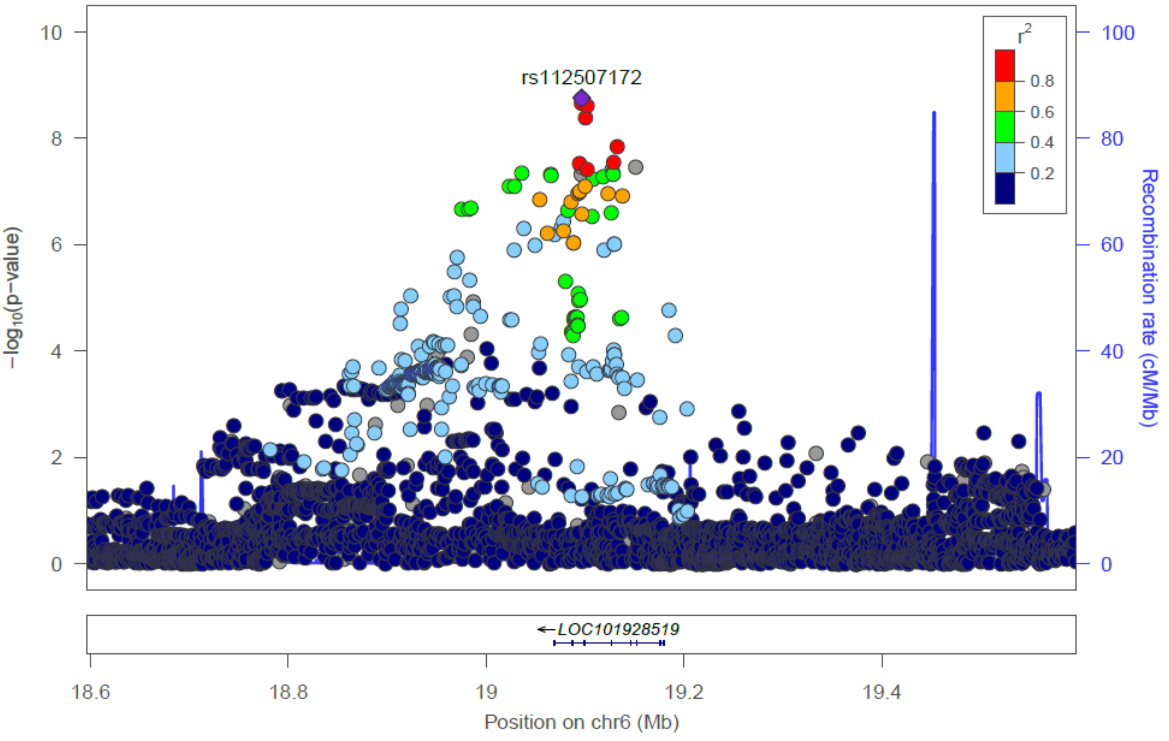
r2
0.8
0.6
0.4
0.2
rs112507172
−log10(p−value)
Recombination rate (cM/Mb)
LOC101928519
18.6
18.8
19
19.2
19.4
Position on chr6 (Mb)

iv)

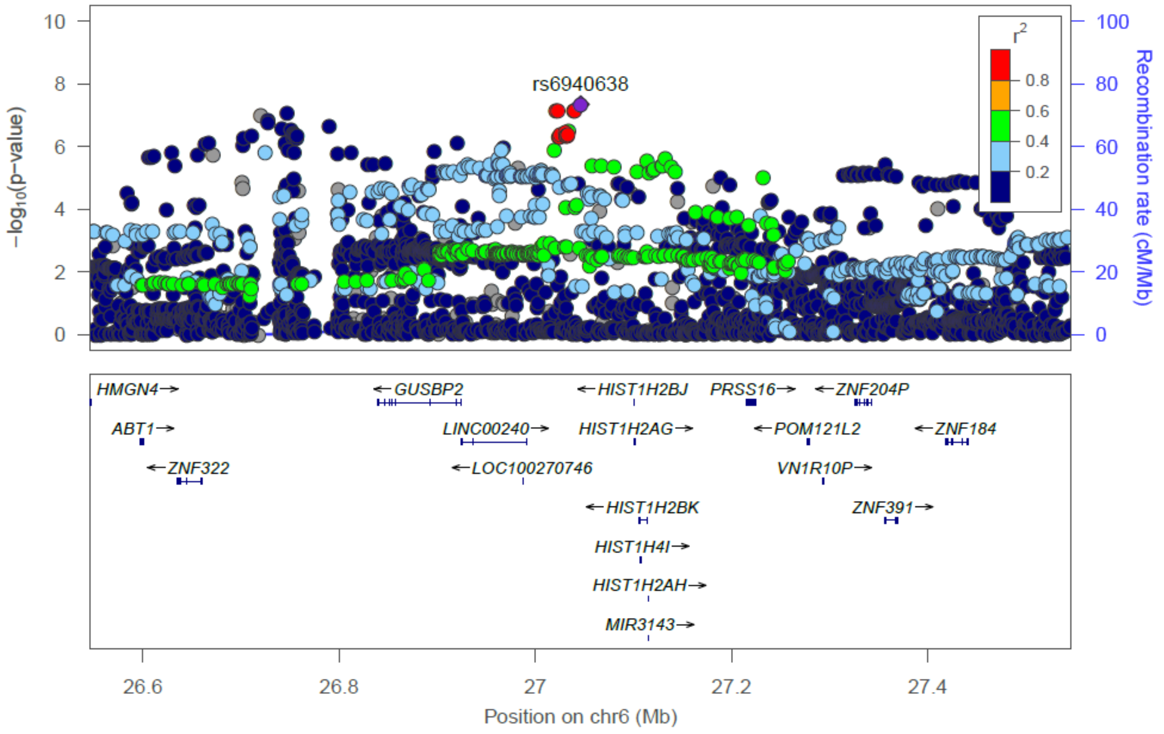

rs6940638
-log10(p-value)
Recombination rate (cM/Mb)
r2
0.8
0.6
0.4
0.2
HMGN4→
ABT1→
←ZNF322
←GUSBP2
LINC00240→
←LOC100270746
←HIST1H2BJ
HIST1H2AG→
←HIST1H2BK
HIST1H4I→
HIST1H2AH→
MIR3143→
PRSS16→
←POM121L2
VN1R10P→
←ZNF204P
←ZNF184
ZNF391→
26.6
26.8
27
27.2
27.4
Position on chr6 (Mb)

v)

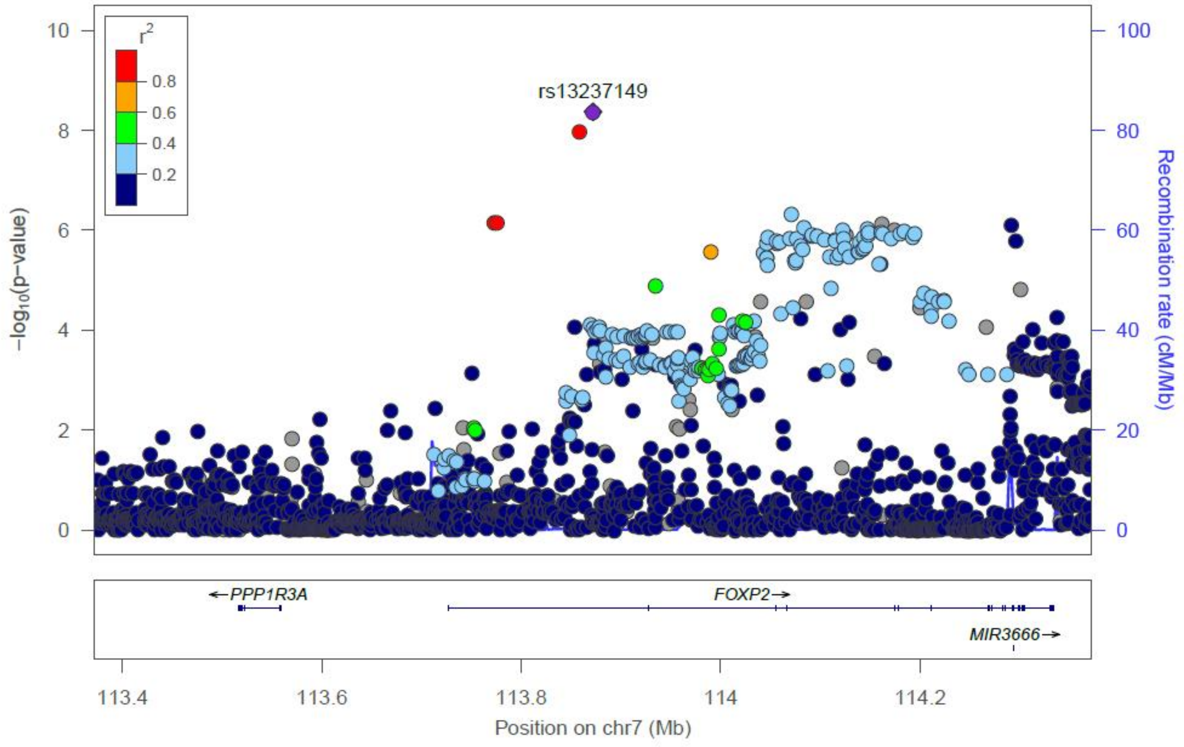

rs13237149
-log10(p-value)
Recombination rate (cM/Mb)
r2
0.8
0.6
0.4
0.2
←PPP1R3A
FOXP2→
MIR3666→
113.4
113.6
113.8
114
114.2
Position on chr7 (Mb)

vi)

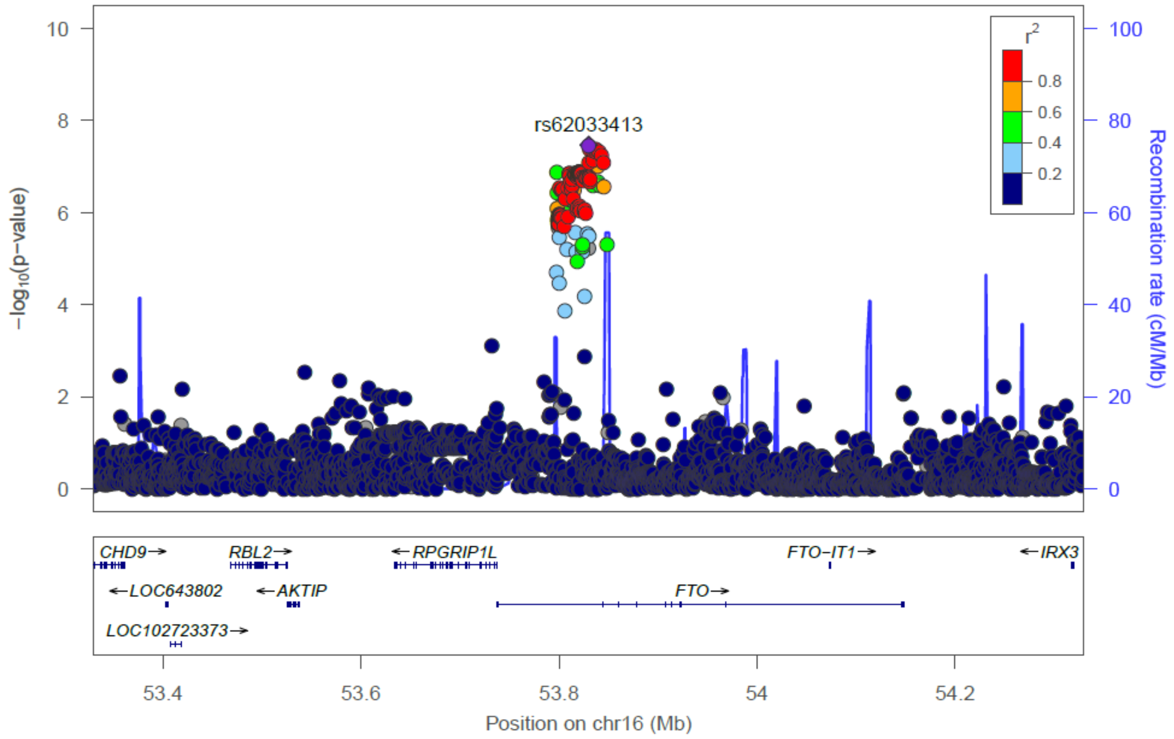

rs62033413
r²
0.8
0.6
0.4
0.2
−log10(p−value)
Recombination rate (cM/Mb)
CHD9→
RBL2→
←RPGRIP1L
FTO−IT1→
←IRX3
←LOC643802
←AKTIP
FTO→
LOC102723373→
53.4
53.6
53.8
54
54.2
Position on chr16 (Mb)

vii)

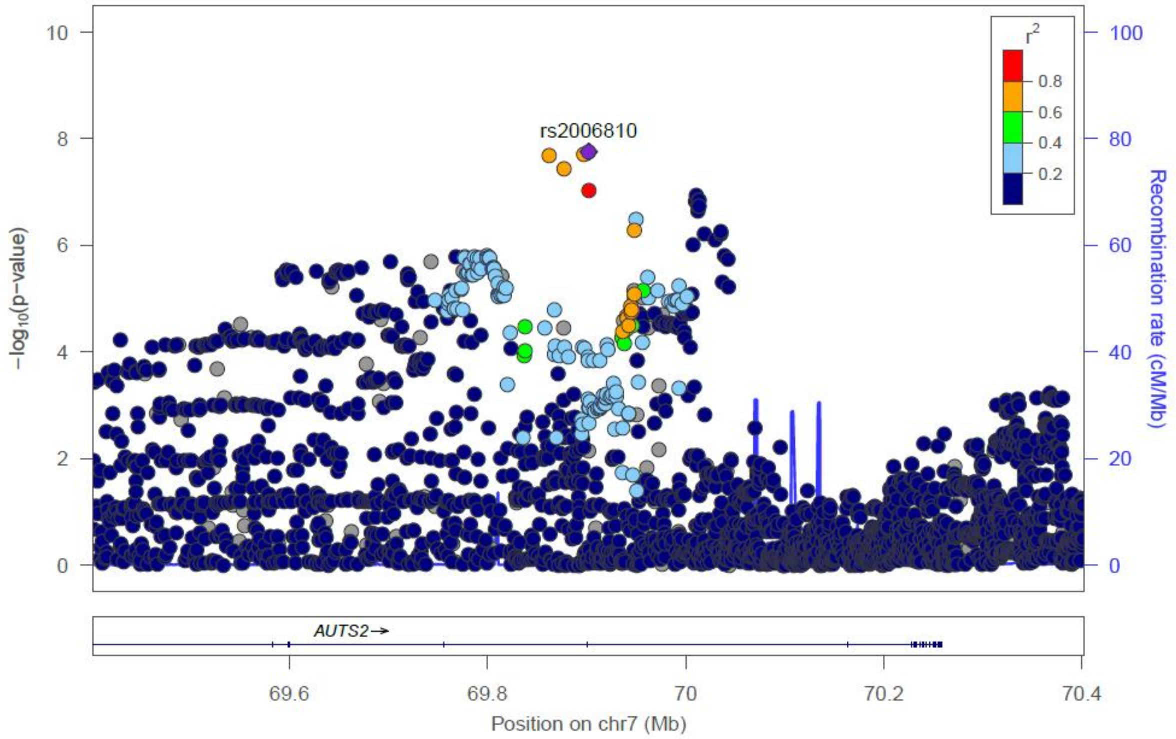

rs2006810
r²
0.8
0.6
0.4
0.2
−log10(p−value)
Recombination rate (cM/Mb)
AUTS2→
69.6
69.8
70
70.2
70.4
Position on chr7 (Mb)

viii)

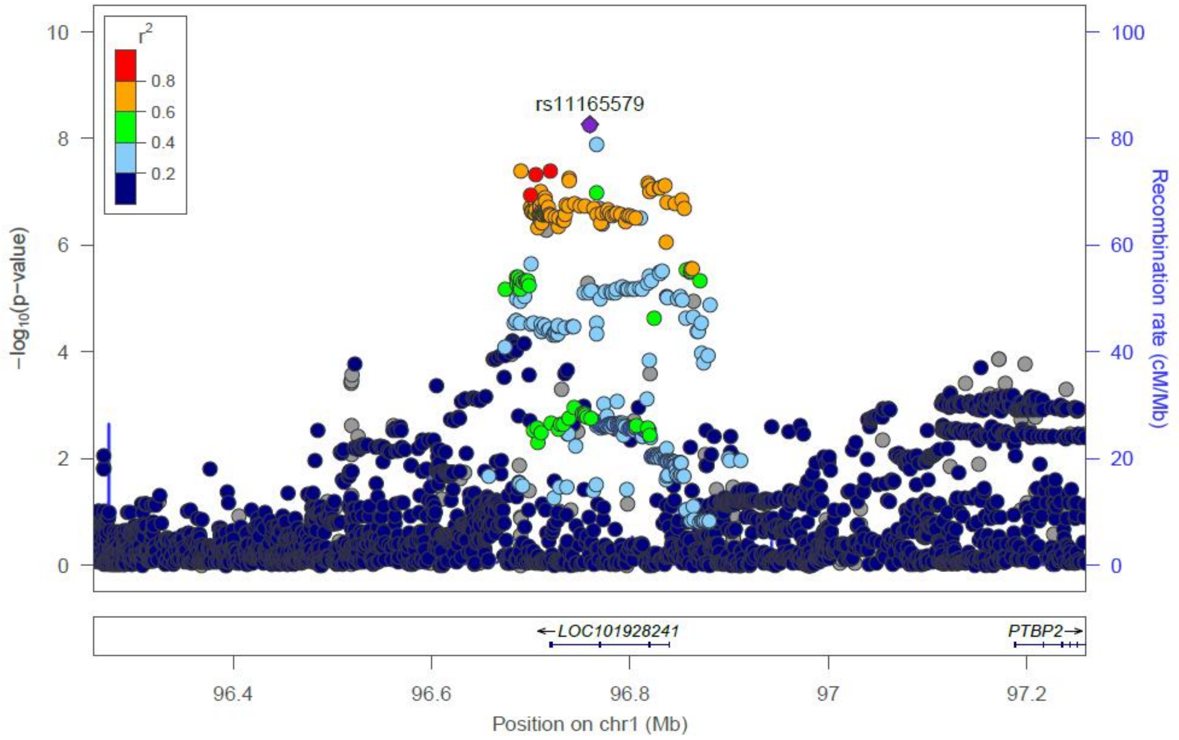

r2
0.8
0.6
0.4
0.2
rs11165579
−log10(p−value)
Recombination rate (cM/Mb)
←LOC101928241
PTBP2→
96.4
96.6
96.8
97
97.2
Position on chr1 (Mb)

ix)

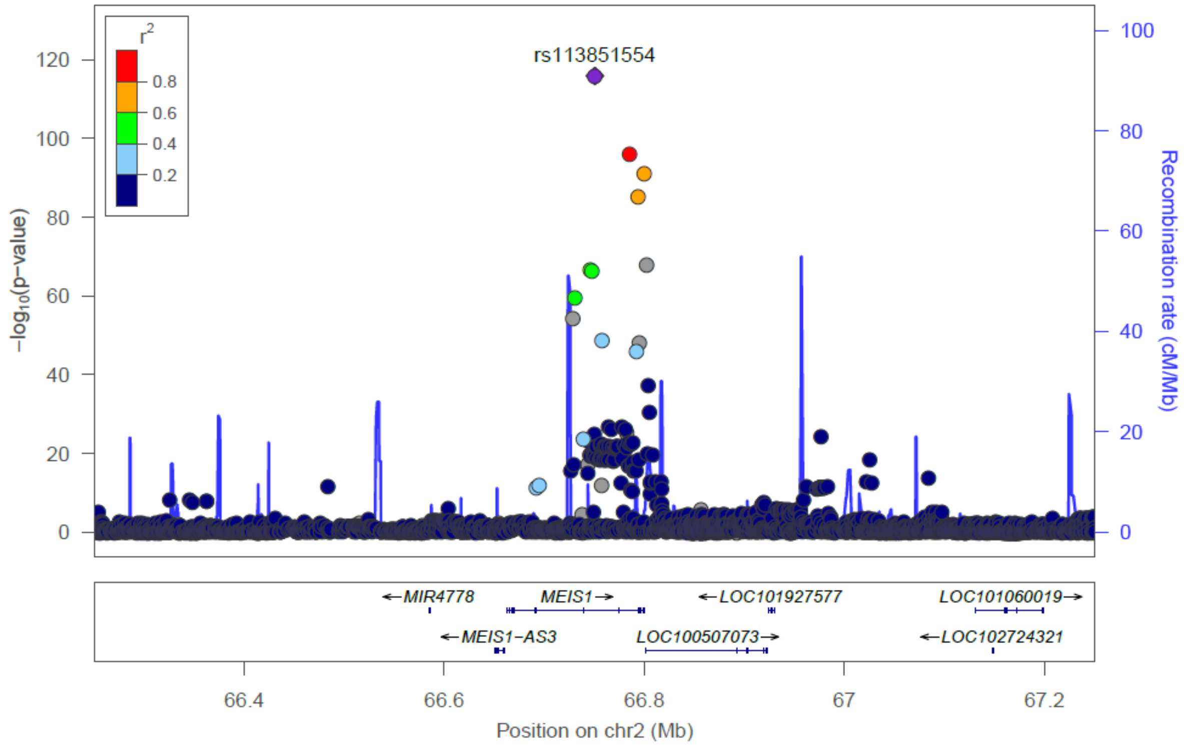

r2
0.8
0.6
0.4
0.2
rs113851554
−log10(p−value)
Recombination rate (cM/Mb)
←MIR4778
MEIS1→
←LOC101927577
LOC101060019→
←MEIS1−AS3
LOC100507073→
←LOC102724321
66.4
66.6
66.8
67
67.2
Position on chr2 (Mb)

x)

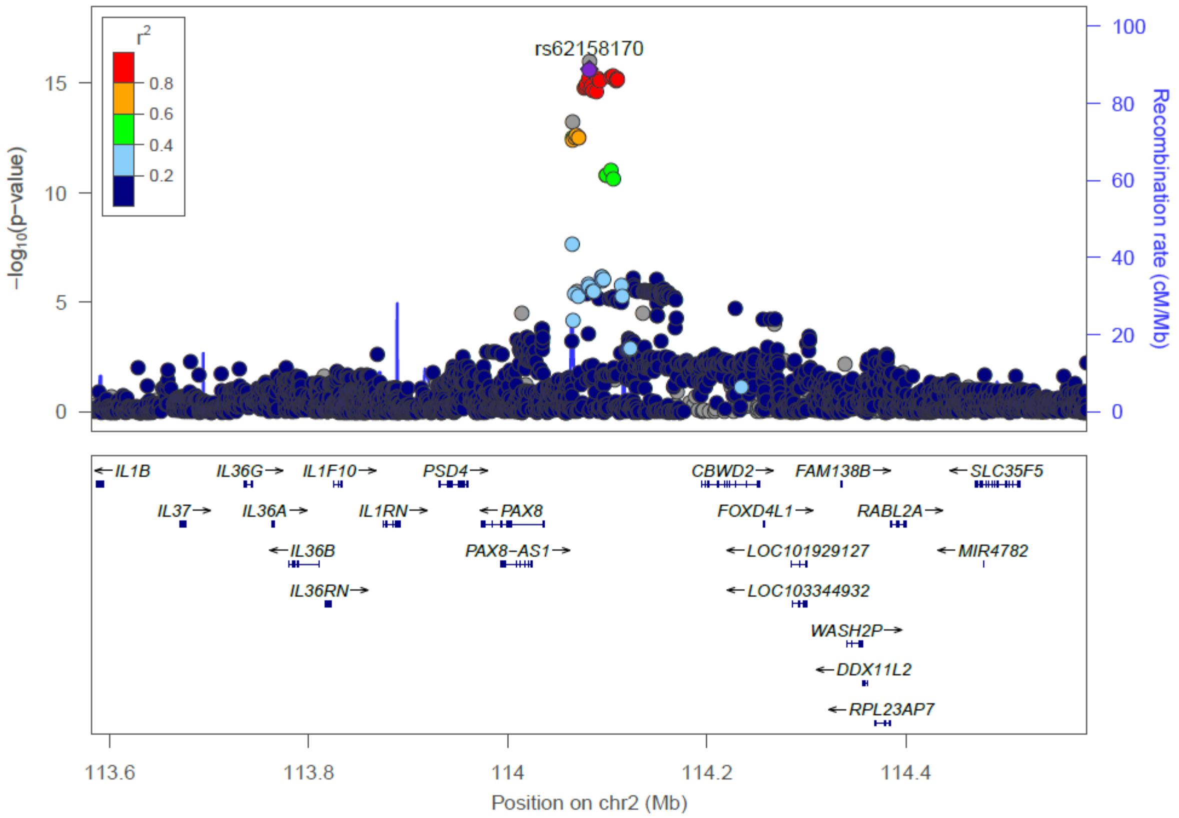
rs62158170
r2
0.8
0.6
0.4
0.2
−log10(p−value)
Recombination rate (cM/Mb)
IL1B
IL36G
IL1F10
PSD4
CBWD2
FAM138B
SLC35F5
IL37
IL36A
IL1RN
PAX8
FOXD4L1
RABL2A
IL36B
PAX8−AS1
LOC101929127
MIR4782
IL36RN
LOC103344932
WASH2P
DDX11L2
RPL23AP7
113.6
113.8
114
114.2
114.4
Position on chr2 (Mb)

xi)

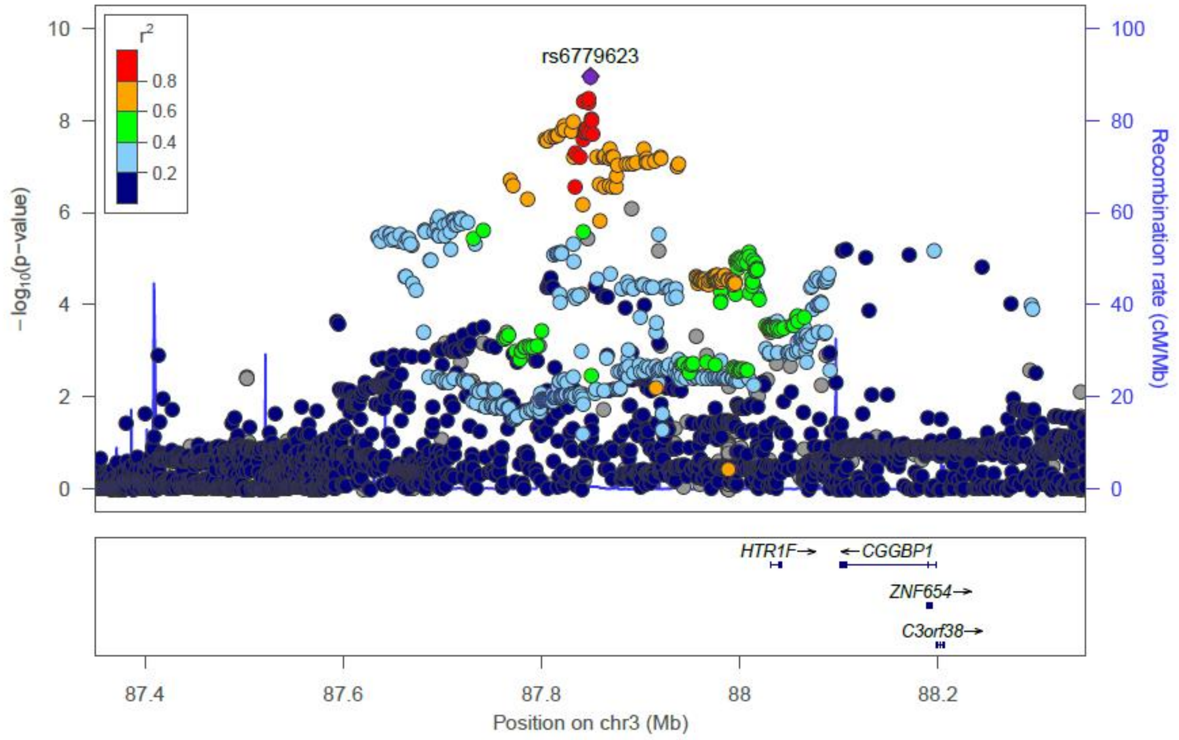
rs6779623
r2
0.8
0.6
0.4
0.2
−log10(p−value)
Recombination rate (cM/Mb)
HTR1F
CGGBP1
ZNF654
C3orf38
87.4
87.6
87.8
88
88.2
Position on chr3 (Mb)

xii)

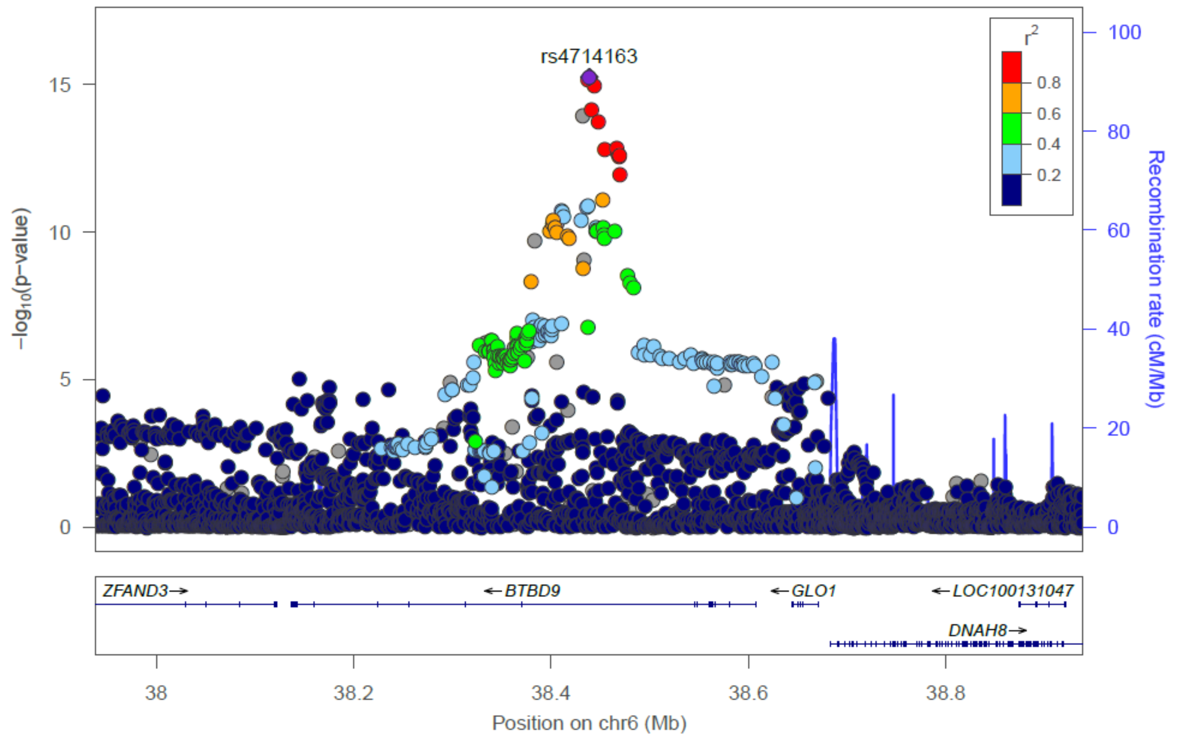
rs4714163
r2
0.8
0.6
0.4
0.2
15
10
5
0
−log10(p−value)
100
80
60
40
20
0
Recombination rate (cM/Mb)
ZFAND3→
←BTBD9
←GLO1
←LOC100131047
DNAH8→
38
38.2
38.4
38.6
38.8
Position on chr6 (Mb)

xiii)

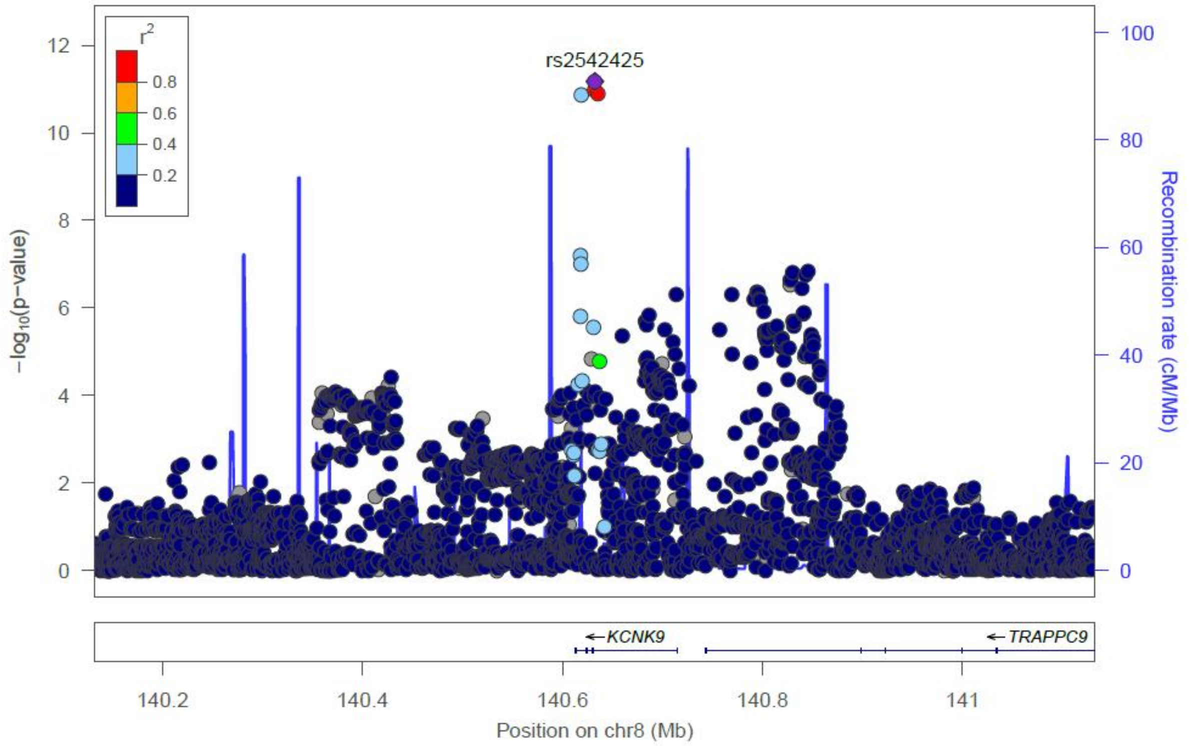
rs2542425
r2
0.8
0.6
0.4
0.2
12
10
8
6
4
2
0
−log10(p−value)
100
80
60
40
20
0
Recombination rate (cM/Mb)
←KCNK9
←TRAPPC9
140.2
140.4
140.6
140.8
141
Position on chr8 (Mb)

xiv)

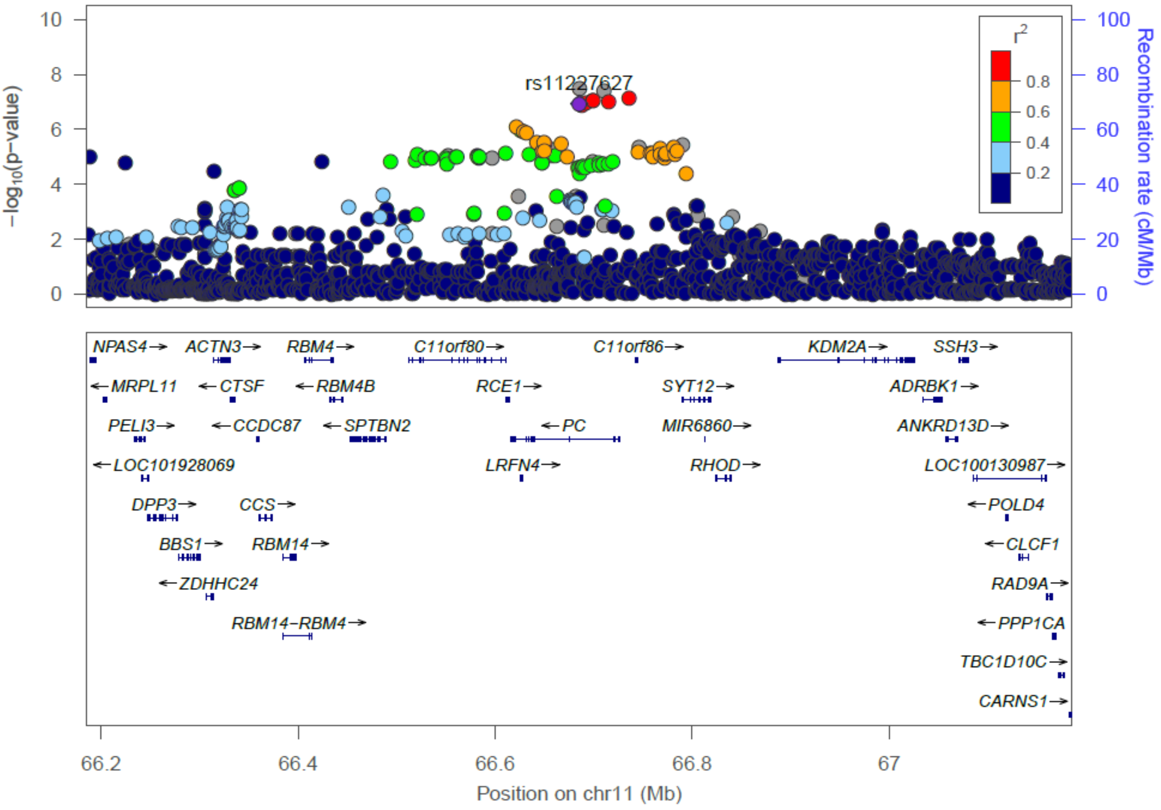

rs11227627
−log10(p−value)
Recombination rate (cM/Mb)
r2
0.8
0.6
0.4
0.2
10
8
6
4
2
0
100
80
60
40
20
0
NPAS4→
ACTN3→
RBM4→
C11orf80→
C11orf86→
KDM2A→
SSH3→
←MRPL11
←CTSF
←RBM4B
RCE1→
SYT12→
ADRBK1→
PELI3→
←CCDC87
←SPTBN2
←PC
MIR6860→
ANKRD13D→
←LOC101928069
LRFN4→
RHOD→
LOC100130987→
DPP3→
CCS→
←POLD4
BBS1→
RBM14→
←CLCF1
←ZDHHC24
RAD9A→
RBM14−RBM4→
←PPP1CA
TBC1D10C→
CARNS1→
66.2
66.4
66.6
66.8
67
Position on chr11 (Mb)

xv)

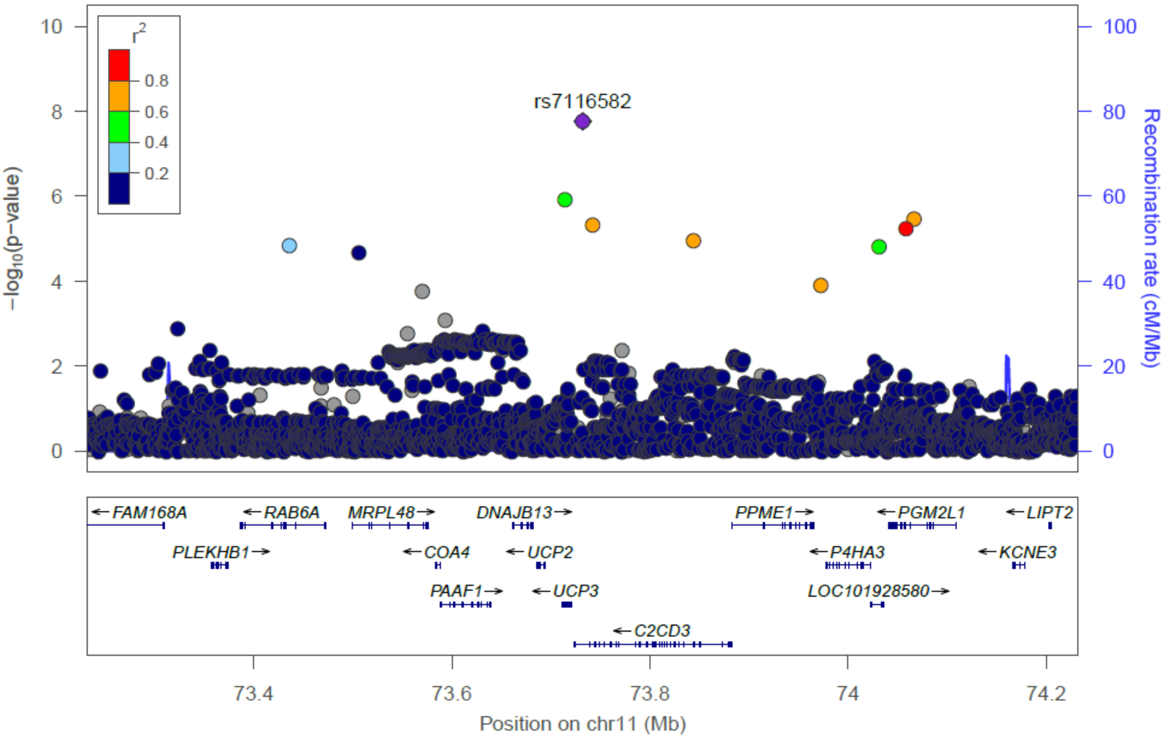

rs7116582
−log10(p−value)
Recombination rate (cM/Mb)
r2
0.8
0.6
0.4
0.2
10
8
6
4
2
0
100
80
60
40
20
0
←FAM168A
←RAB6A
MRPL48→
DNAJB13→
PPME1→
←PGM2L1
←LIPT2
PLEKHB1→
←COA4
←UCP2
←P4HA3
←KCNE3
PAAF1→
←UCP3
LOC101928580→
←C2CD3
73.4
73.6
73.8
74
74.2
Position on chr11 (Mb)

xvi)

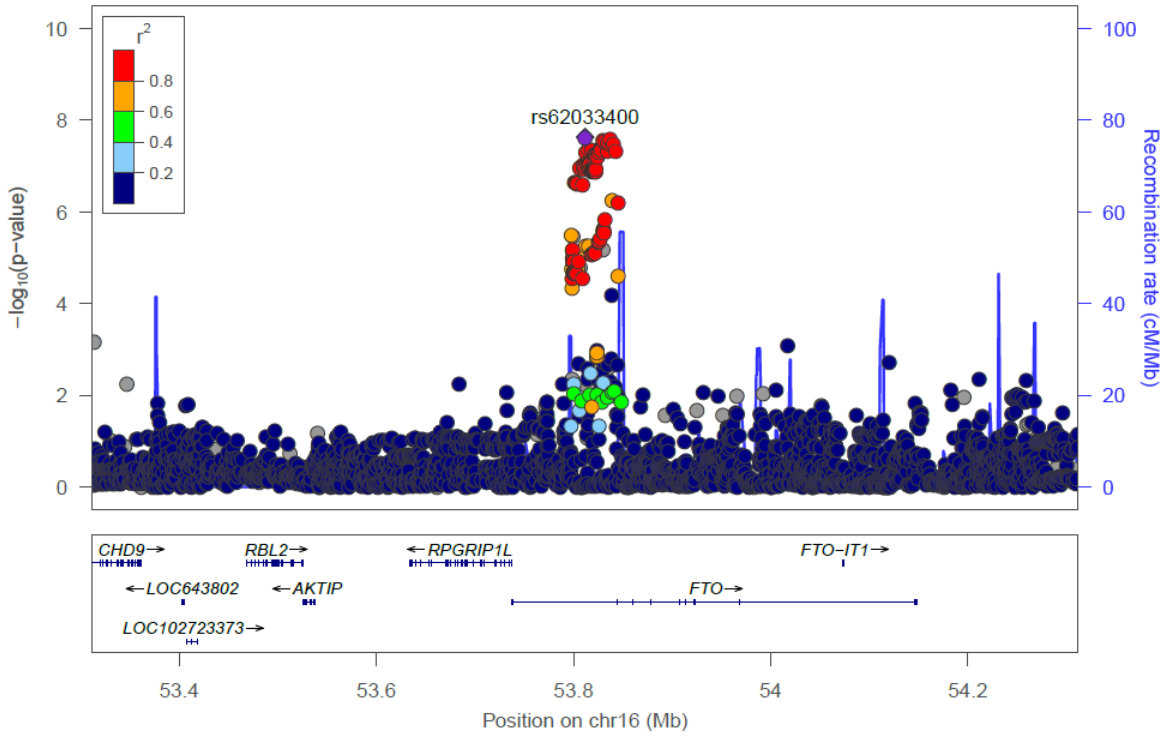
r2
0.8
0.6
0.4
0.2
rs62033400
−log10(p−value)
Recombination rate (cM/Mb)
CHD9→
RBL2→
←RPGRIP1L
FTO−IT1→
←LOC643802
←AKTIP
FTO→
LOC102723373→
53.4
53.6
53.8
54
54.2
Position on chr16 (Mb)

xvii)

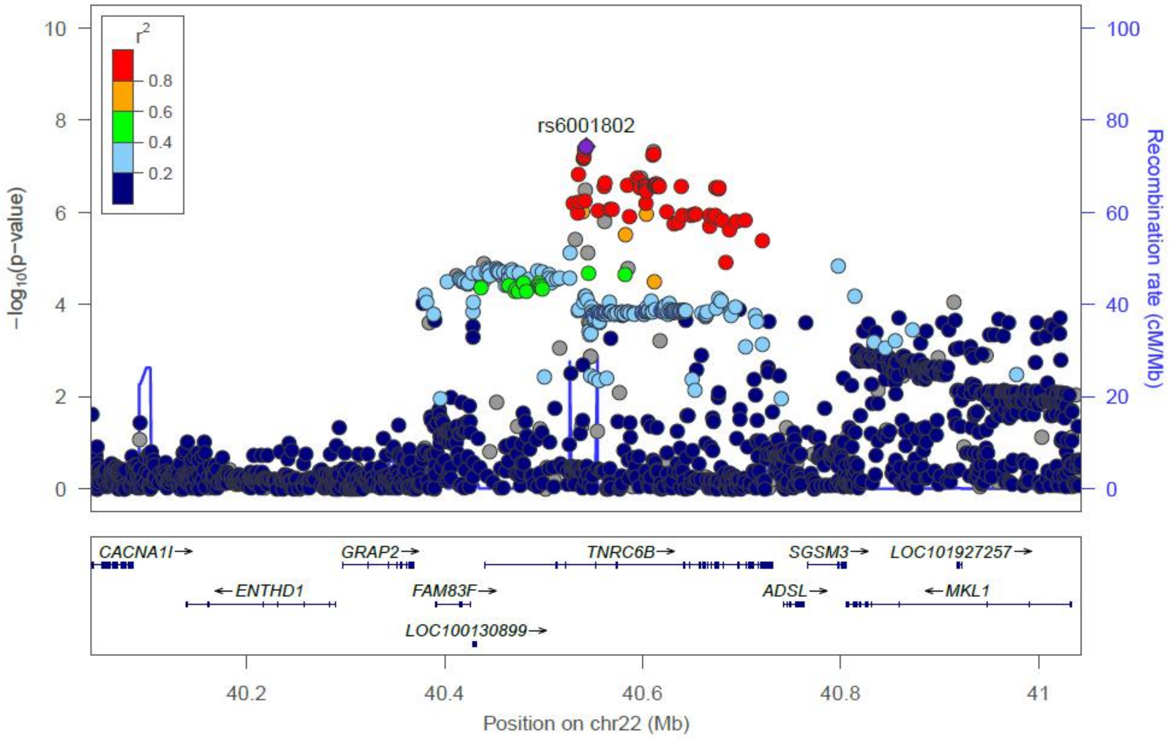
r2
0.8
0.6
0.4
0.2
rs6001802
−log10(p−value)
Recombination rate (cM/Mb)
CACNA1I→
GRAP2→
TNRC6B→
SGSM3→
LOC101927257→
←ENTHD1
FAM83F→
ADSL→
←MKL1
LOC100130899→
40.2
40.4
40.6
40.8
41
Position on chr22 (Mb)

xviii)

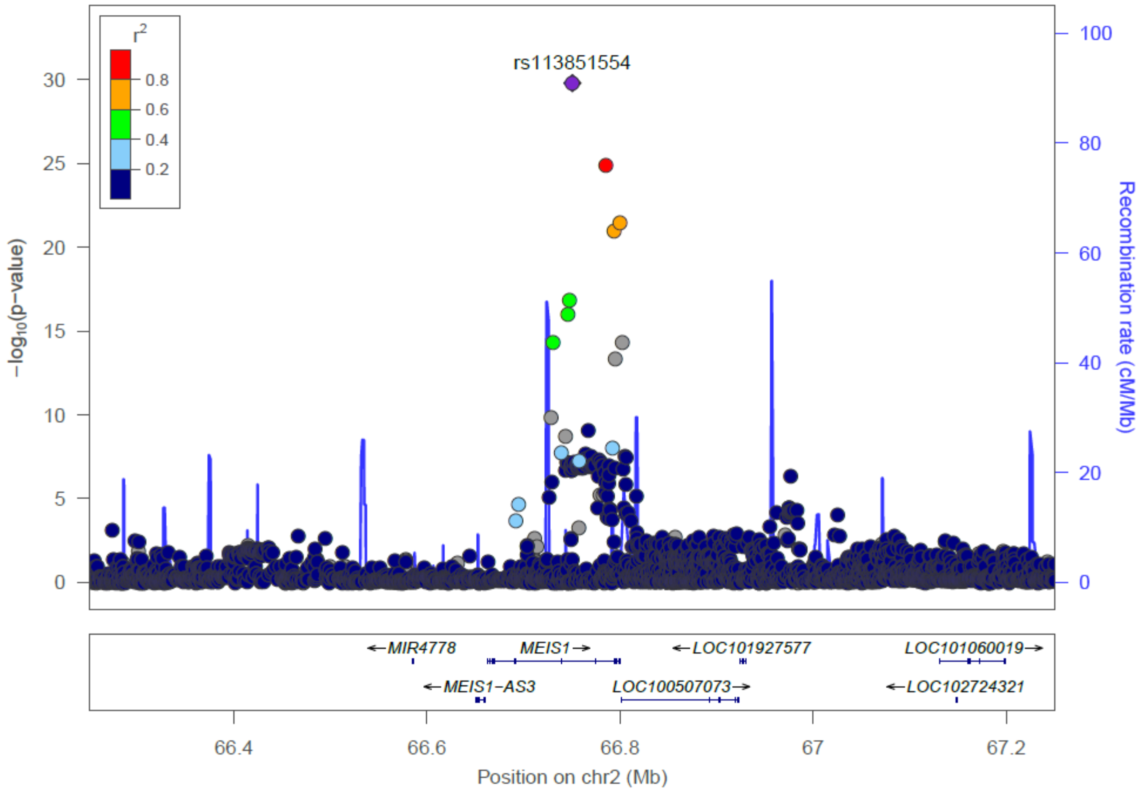

rs113851554
r2
0.8
0.6
0.4
0.2
−log10(p−value)
Recombination rate (cM/Mb)
MIR4778
MEIS1
LOC101927577
LOC101060019
MEIS1−AS3
LOC100507073
LOC102724321
66.4
66.6
66.8
67
67.2
Position on chr2 (Mb)

xix)

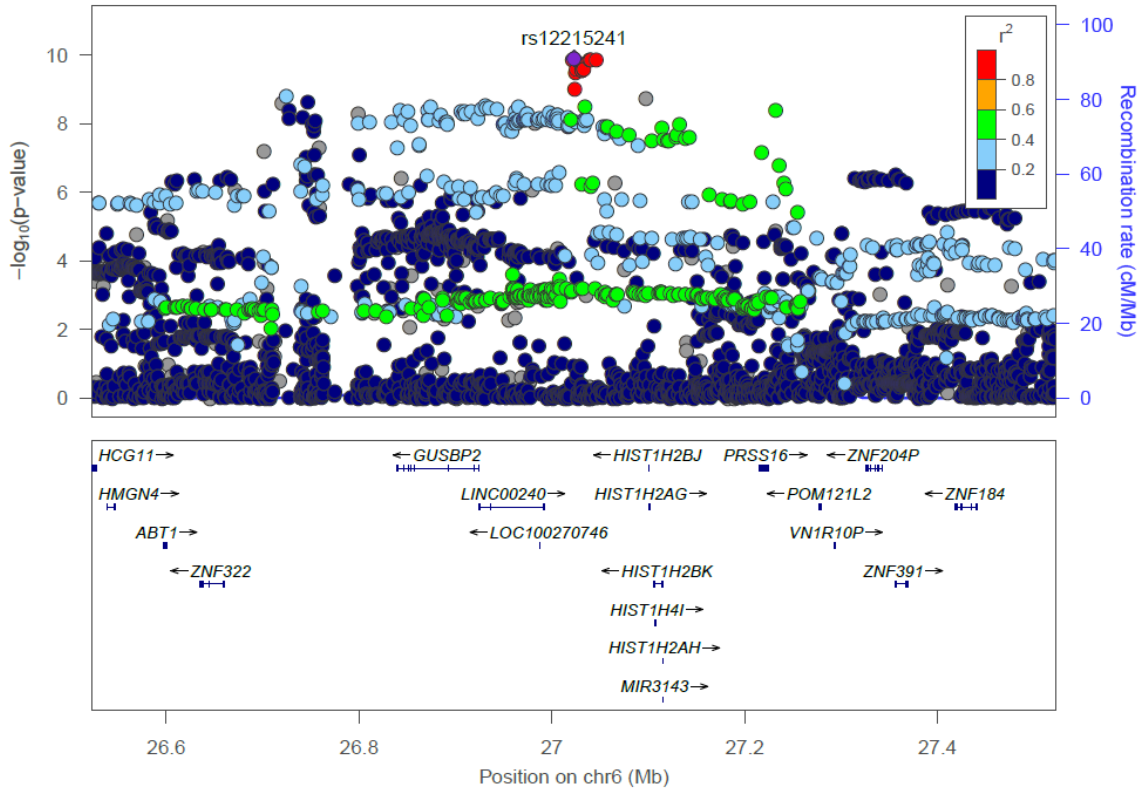

rs12215241
r2
0.8
0.6
0.4
0.2
−log10(p−value)
Recombination rate (cM/Mb)
HCG11
GUSBP2
HIST1H2BJ
PRSS16
ZNF204P
HMGN4
LINC00240
HIST1H2AG
POM121L2
ZNF184
ABT1
LOC100270746
VN1R10P
ZNF322
HIST1H2BK
ZNF391
HIST1H4I
HIST1H2AH
MIR3143
26.6
26.8
27
27.2
27.4
Position on chr6 (Mb)

xx)

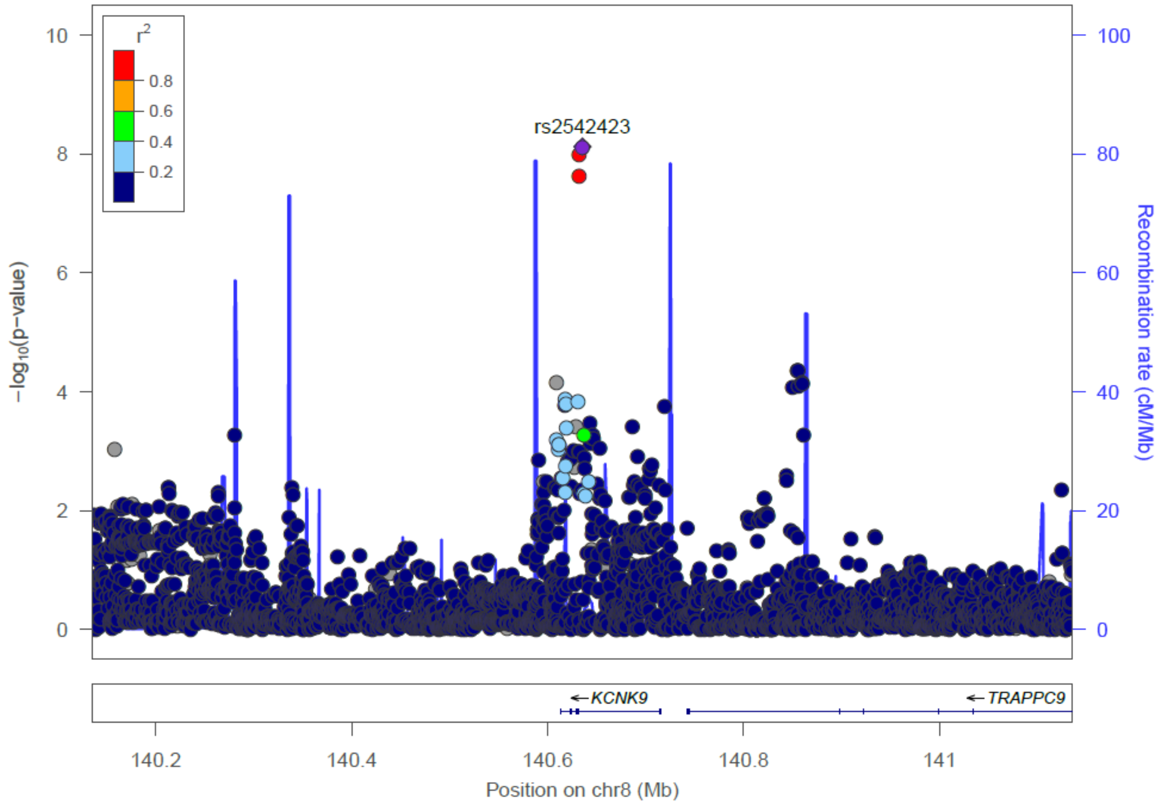

rs2542423
r2
0.8
0.6
0.4
0.2
−log10(p−value)
Recombination rate (cM/Mb)
KCNK9
TRAPPC9
140.2
140.4
140.6
140.8
141
Position on chr8 (Mb)

xxi)

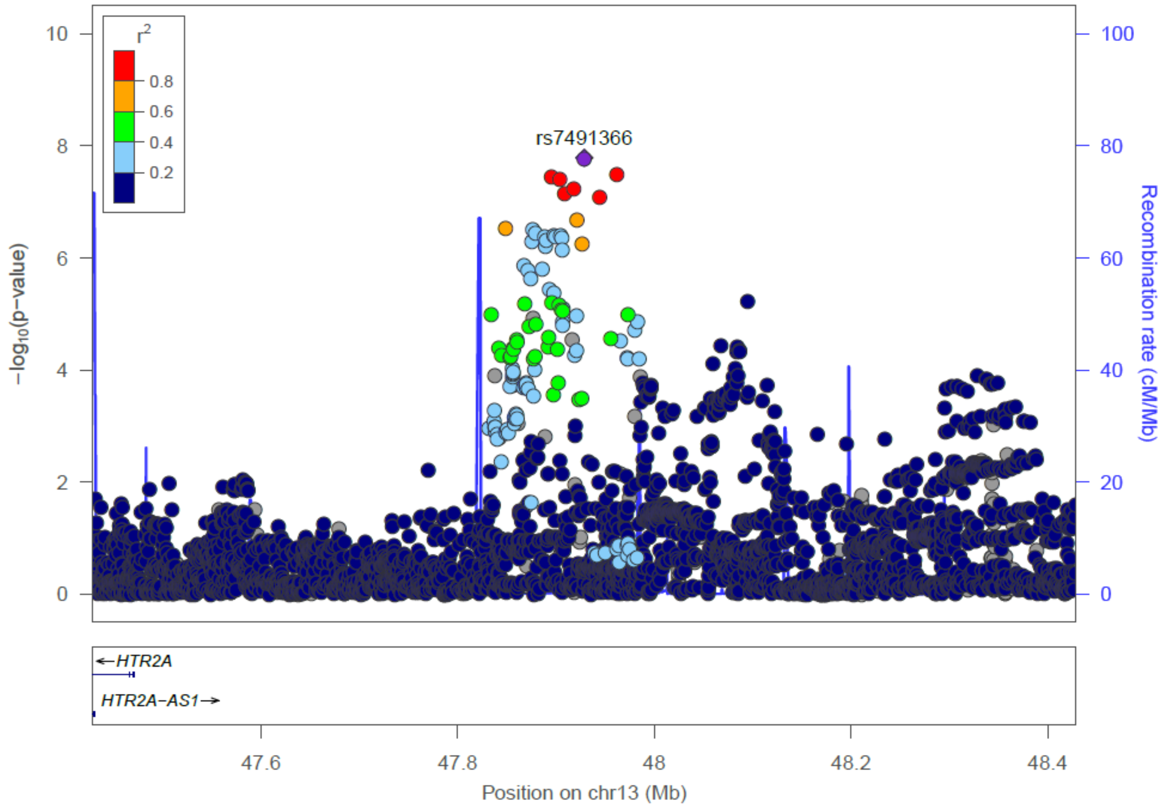

rs7491366
r2
0.8
0.6
0.4
0.2
−log10(p−value)
Recombination rate (cM/Mb)
HTR2A
HTR2A−AS1
47.6
47.8
48
48.2
48.4
Position on chr13 (Mb)

xxii)

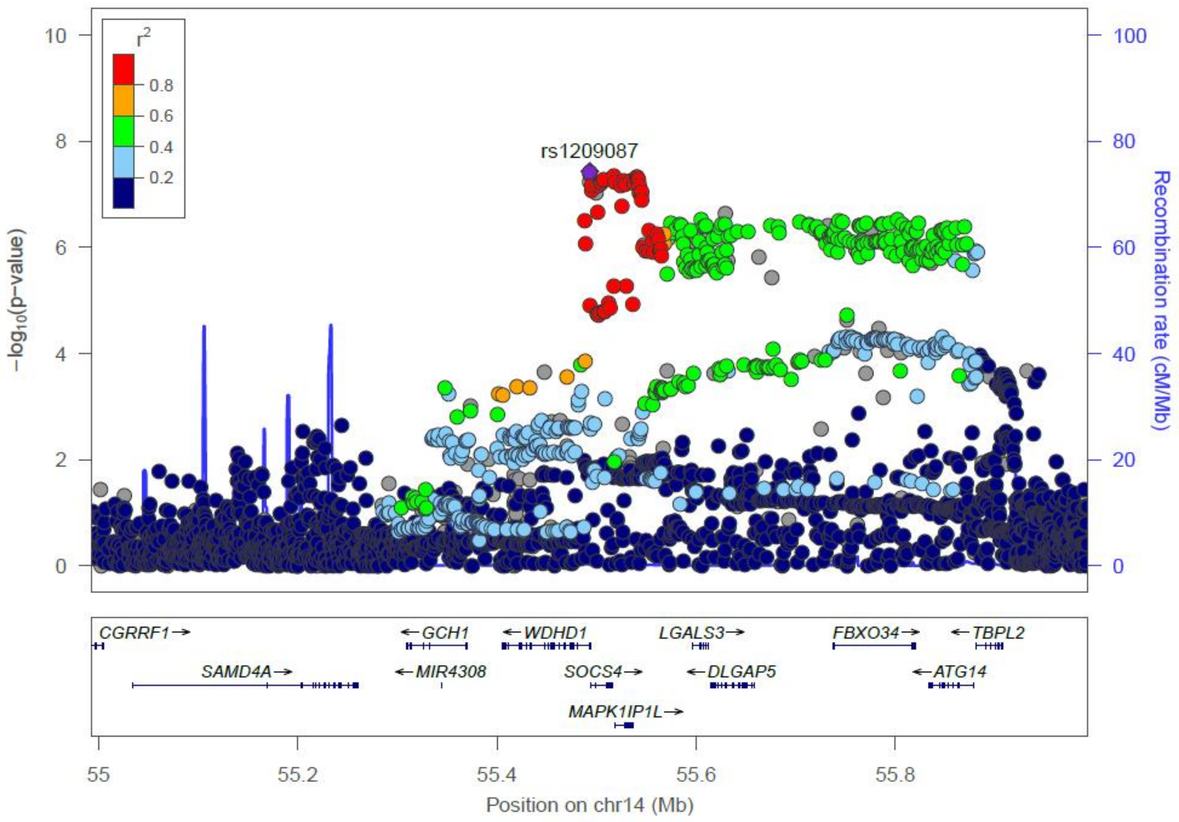
rs1209087
r2
0.8
0.6
0.4
0.2
−log10(p-value)
Recombination rate (cM/Mb)
CGRRF1→
SAMD4A→
←GCH1
←MIR4308
←WDHD1
SOCS4→
MAPK1IP1L→
LGALS3→
←DLGAP5
FBXO34→
←ATG14
←TBPL2
Position on chr14 (Mb)

xxiii)

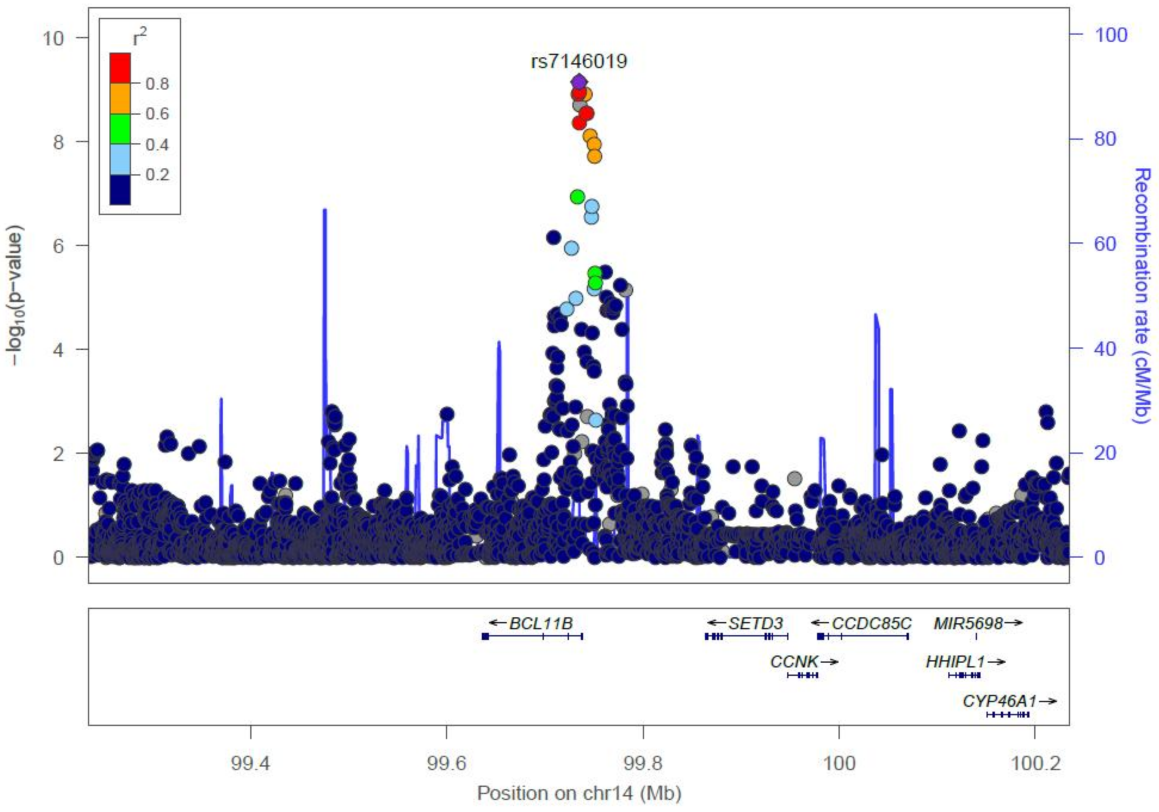
rs7146019
r2
0.8
0.6
0.4
0.2
−log10(p-value)
Recombination rate (cM/Mb)
←BCL11B
←SETD3
CCNK→
←CCDC85C
MIR5698→
HHIPL1→
CYP46A1→
Position on chr14 (Mb)

xxiv)

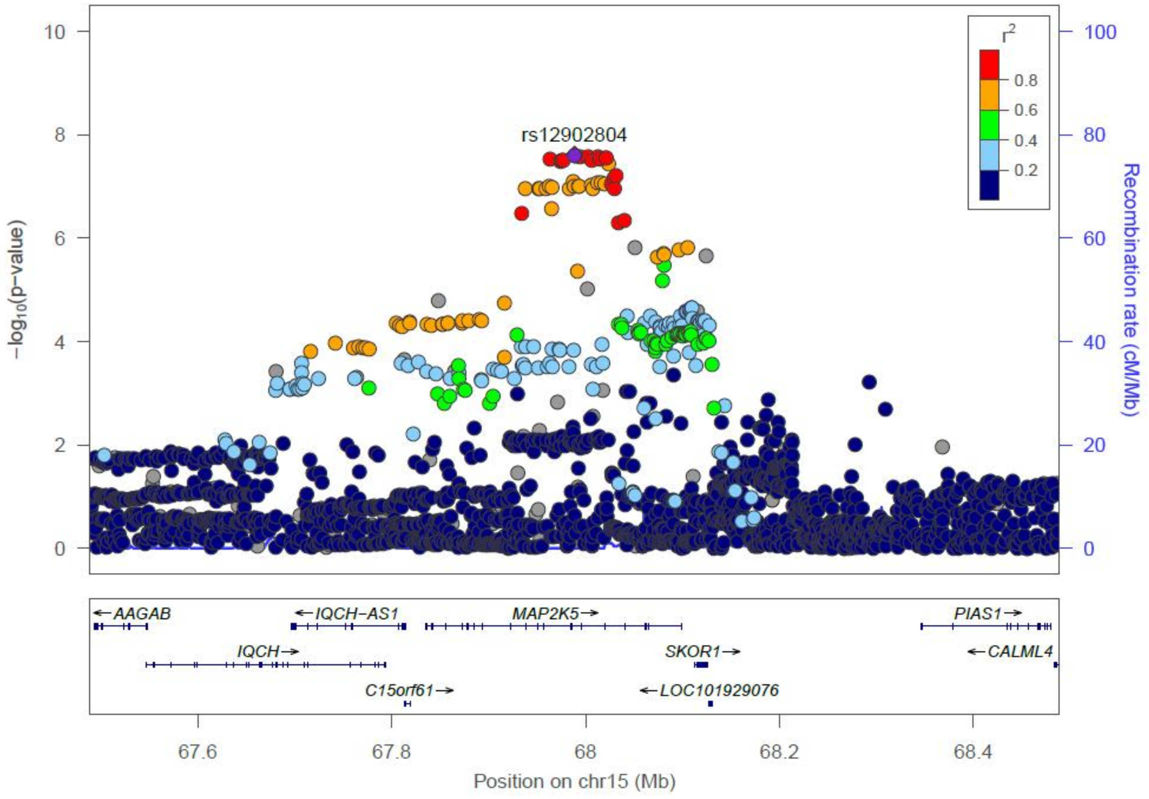
rs12902804
r²
0.8
0.6
0.4
0.2
−log10(p−value)
Recombination rate (cM/Mb)
AAGAB
IQCH−AS1
MAP2K5
PIAS1
IQCH
SKOR1
CALML4
C15orf61
LOC101929076
67.6
67.8
68
68.2
68.4
Position on chr15 (Mb)

xxv)

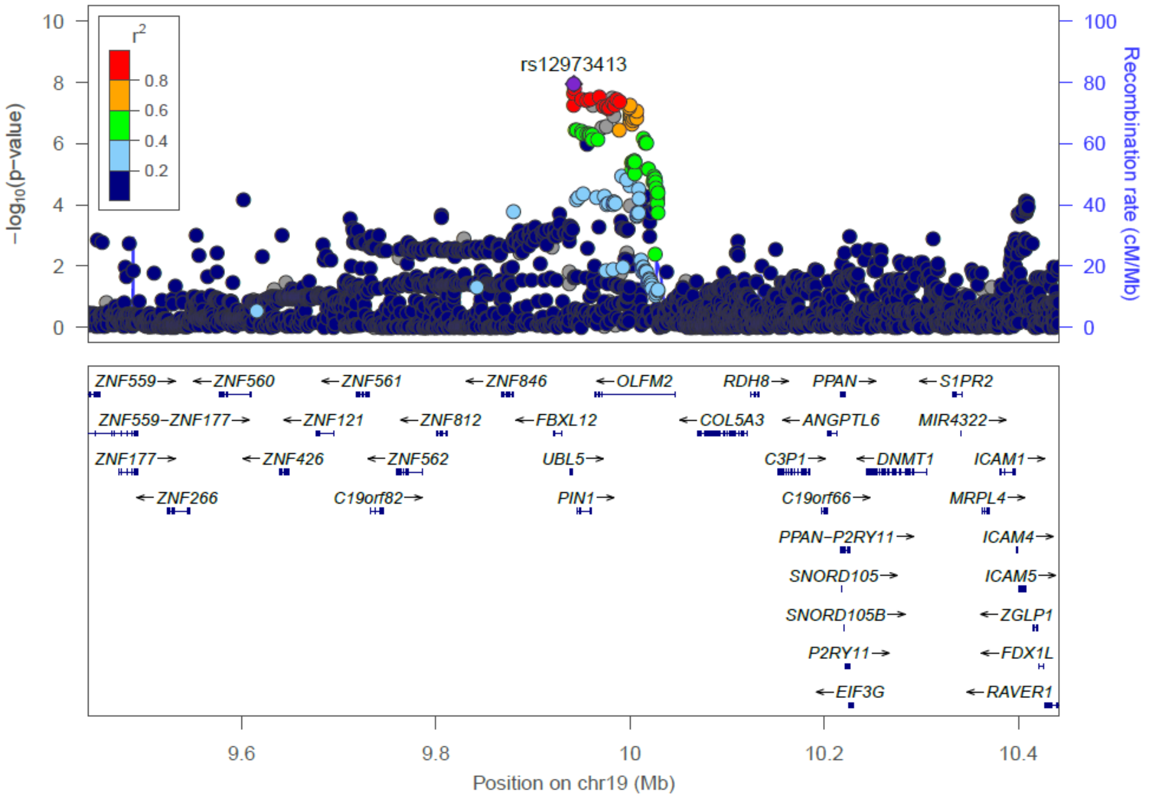
rs12973413
r²
0.8
0.6
0.4
0.2
−log10(p−value)
Recombination rate (cM/Mb)
ZNF559
ZNF560
ZNF561
ZNF846
OLFM2
RDH8
PPAN
S1PR2
ZNF559−ZNF177
ZNF121
ZNF812
FBXL12
COL5A3
ANGPTL6
MIR4322
ZNF177
ZNF426
ZNF562
UBL5
C3P1
DNMT1
ICAM1
ZNF266
C19orf82
PIN1
C19orf66
MRPL4
PPAN−P2RY11
ICAM4
SNORD105
ICAM5
SNORD105B
ZGLP1
P2RY11
FDX1L
EIF3G
RAVER1
9.6
9.8
10
10.2
10.4
Position on chr19 (Mb)

Supplementary Figure 7. Comparison of effect size estimates (β) between the main GWAS (N = 80,013) and the restricted sample excluding individuals with <7 hours of night-time sleep (N = 34,908) for: (a) night-time sleep duration, (b) sleep efficiency, (c) REM sleep, and (d) NREM sleep. Red dots highlight variants reaching genome-wide significance ($P < 5\times10^{-8}$) in the full dataset. The diagonal dashed line represents the identity line (y = x), while the green line is the regression fit.

a)

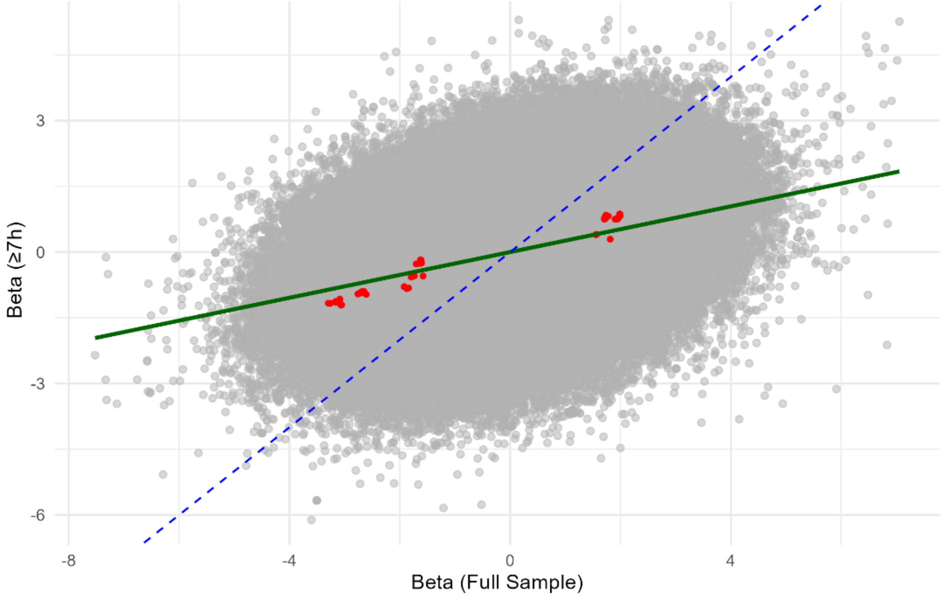

b)

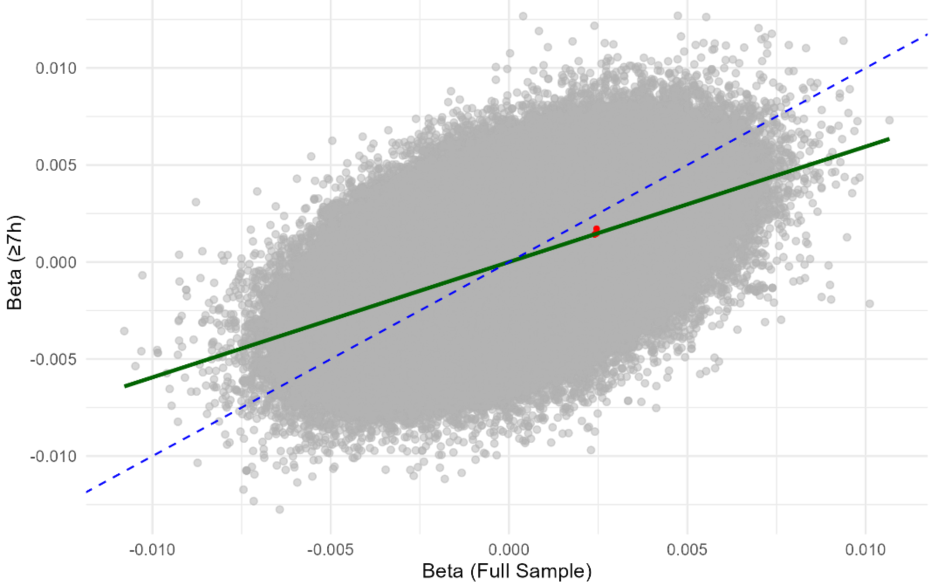

c)

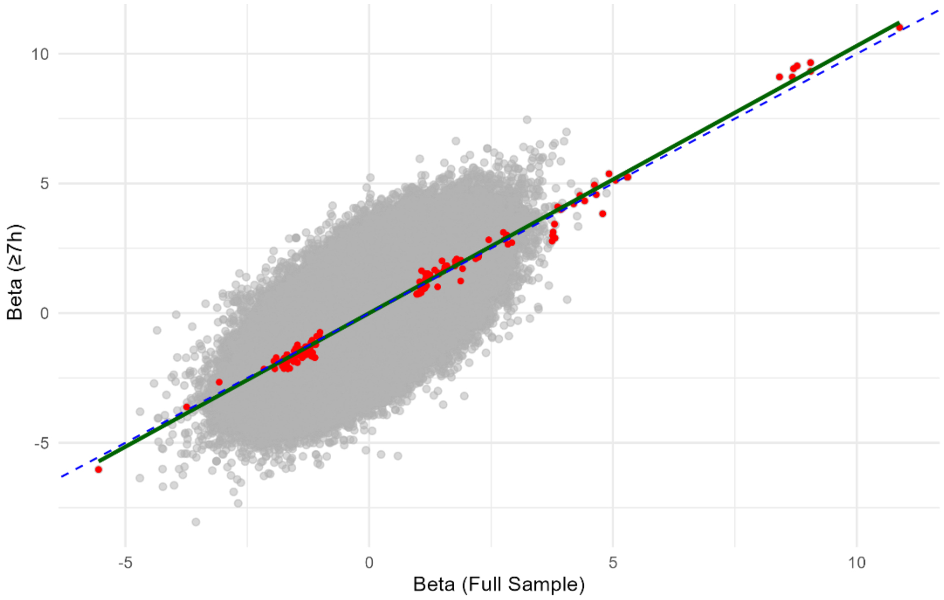

10
5
0
-5
Beta (≥7h)
-5
0
5
10
Beta (Full Sample)

d)

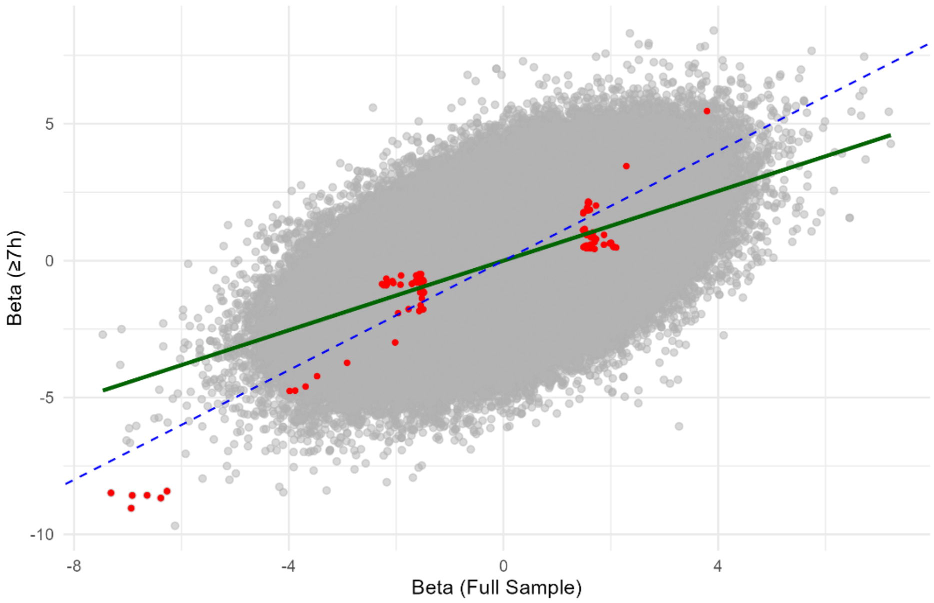

5
0
-5
-10
Beta (≥7h)
-8
-4
0
4
Beta (Full Sample)

Supplementary Figure 8. Comparison of standard errors (SE) between the main GWAS (N = 80,013) and the restricted sample excluding individuals with <7 hours of night-time sleep (N = 34,908) for: (a) night-time sleep duration, (b) sleep efficiency, (c) REM sleep, and (d) NREM sleep. Red dots denote genome-wide significant variants in the full dataset. The diagonal dashed line represents the identity line (y = x), while the green line is the regression fit.

a)

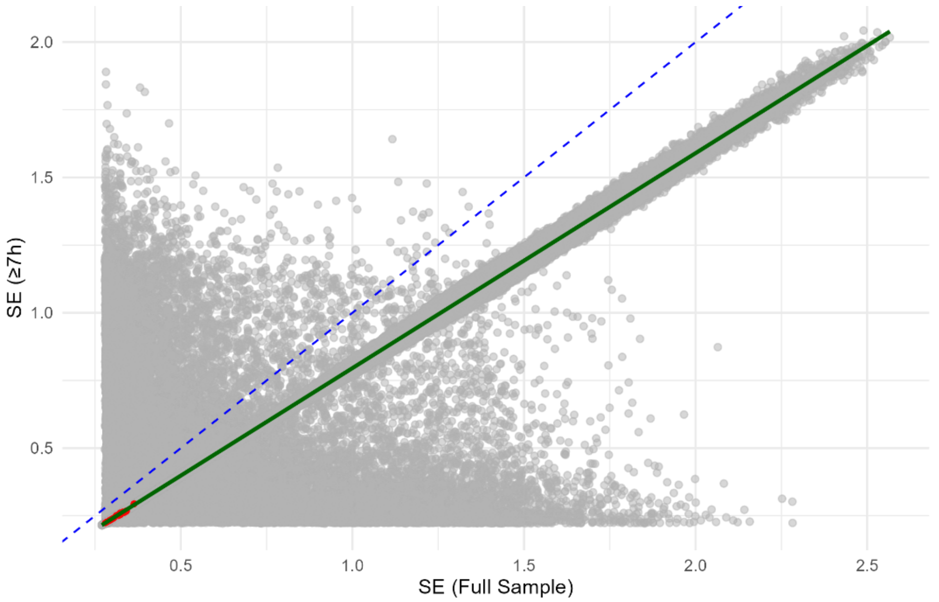

b)

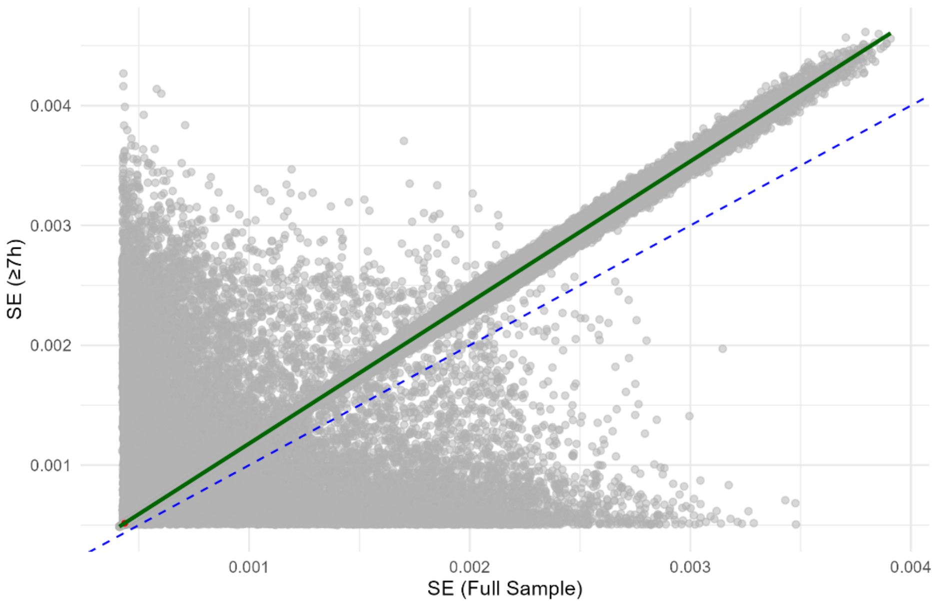

c)

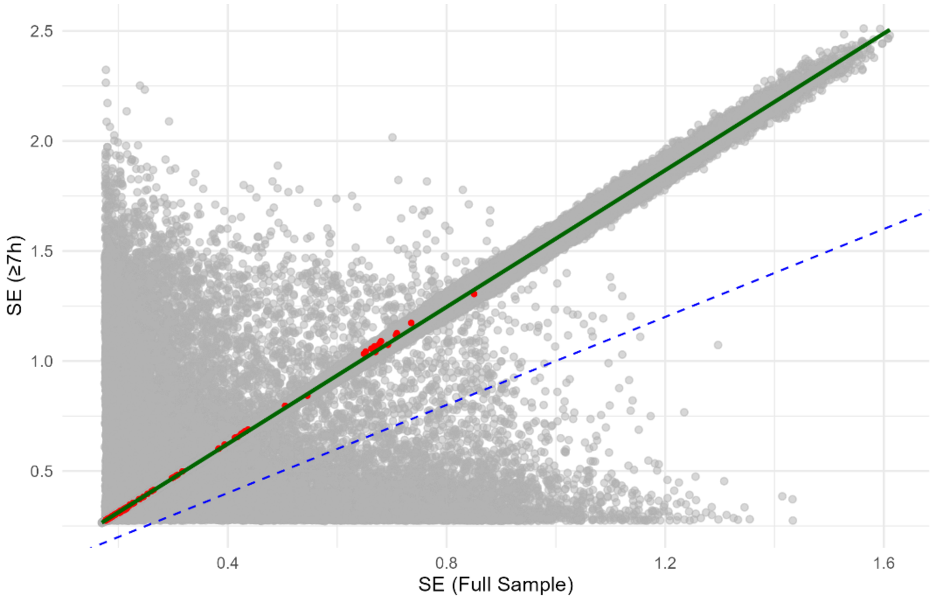
2.5
2.0
1.5
1.0
0.5
SE (≥7h)
0.4
0.8
1.2
1.6
SE (Full Sample)

d)

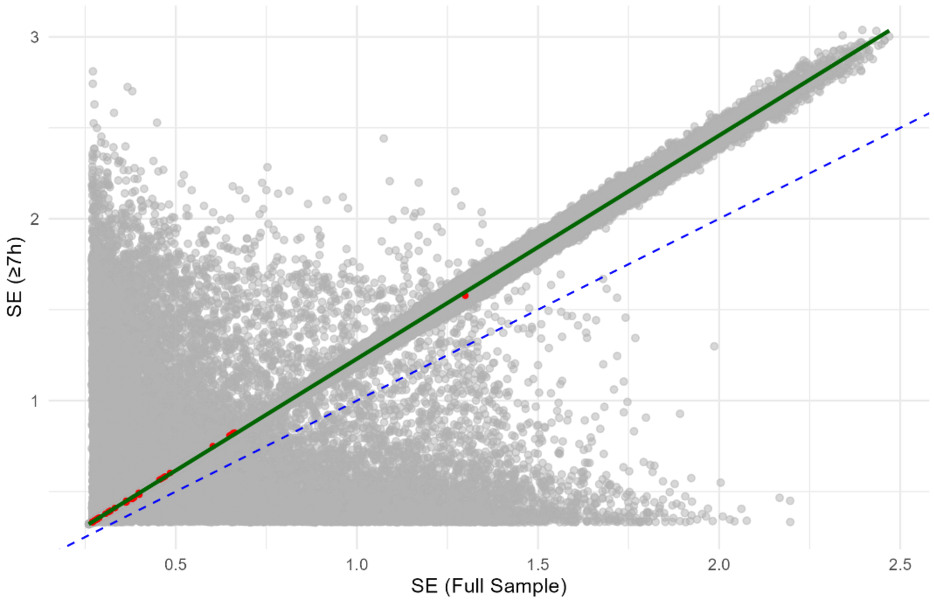
3
2
1
SE (≥7h)
0.5
1.0
1.5
2.0
2.5
SE (Full Sample)

Supplementary Figure 9. Manhattan plot for the overall sample and by sex of sleep traits: a) night-time sleep duration, b) sleep efficiency, c) REM sleep duration, and d) Non-REM sleep duration.

a)

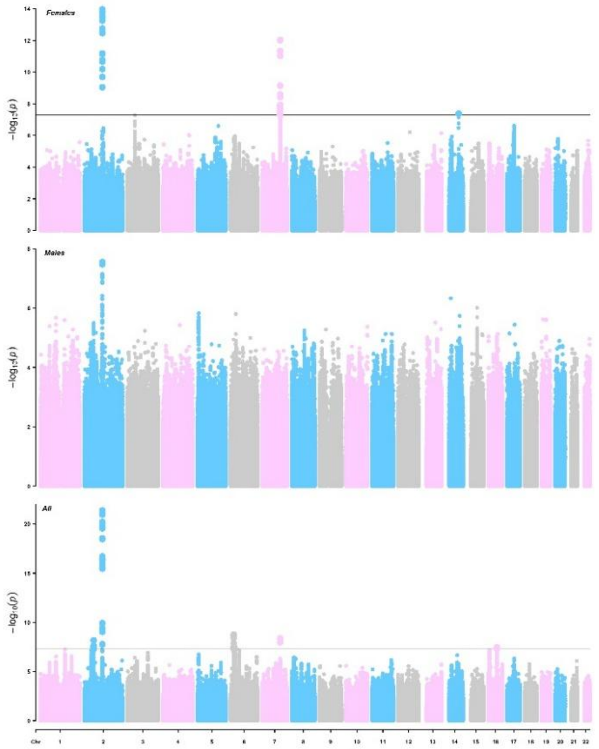

b)

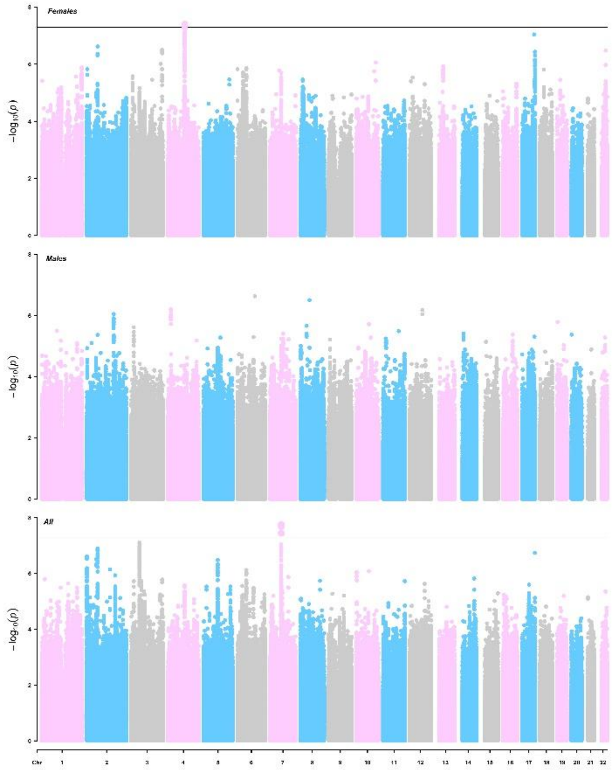

c)

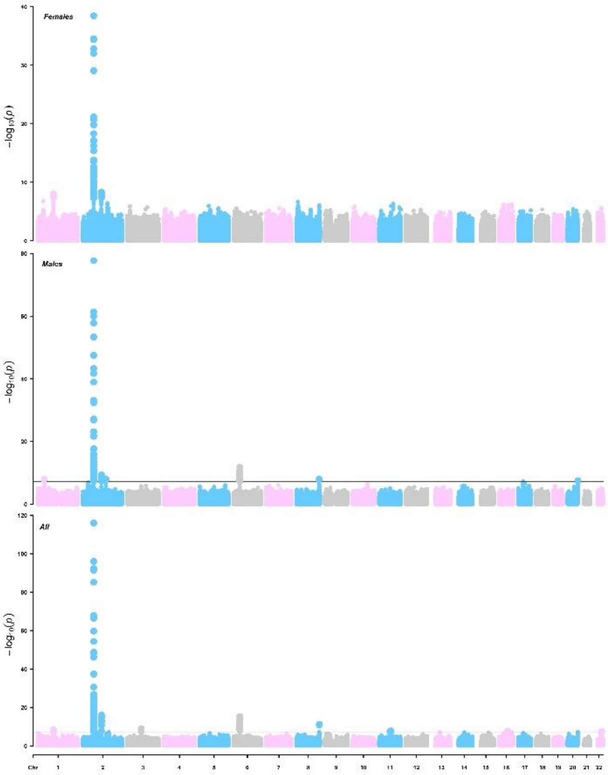
Females
Males
All
$-\log_{10}(p)$
Chr
1
2
3
4
5
6
7
8
9
10
11
12
13
14
15
16
17
18
19
20
21
22

d)

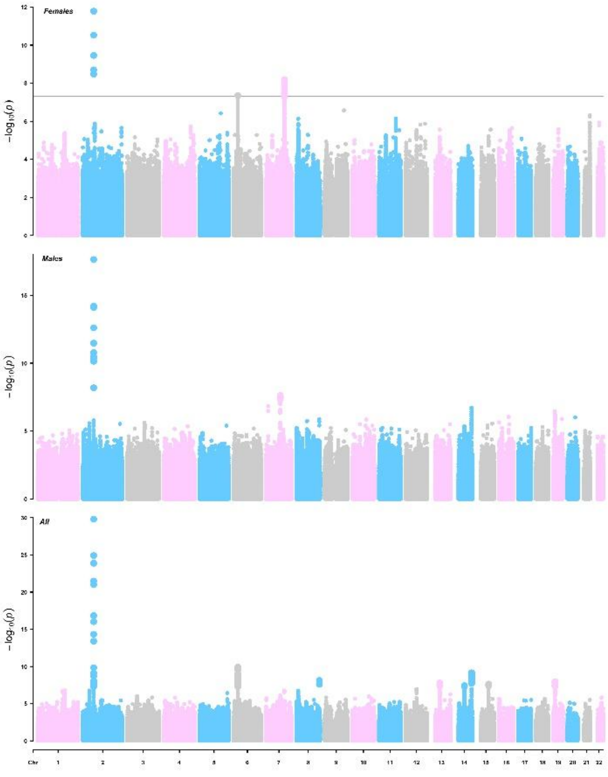
Females
Males
All
$-\log_{10}(p)$
Chr
1
2
3
4
5
6
7
8
9
10
11
12
13
14
15
16
17
18
19
20
21
22

Supplementary Figure 10. Tissue enrichment of genes at associated loci for accelerometer-based sleep traits: a) night-time sleep duration; b) sleep efficiency; c) REM sleep; d) Non-REM sleep. Genes were identified using MAGMA and then analysed for enrichment using the GTEx RNA-seq database (v8) to investigate expression across 30 general tissues and 53 tissue types.

a)

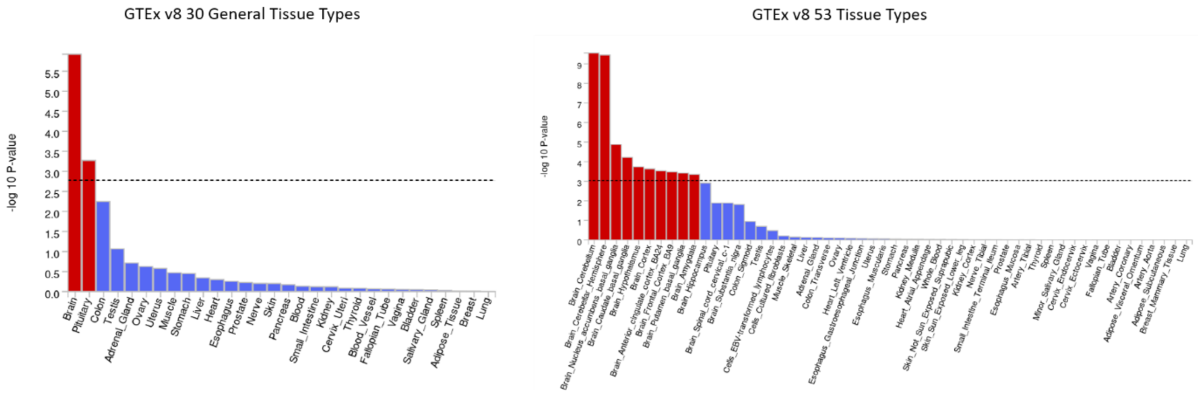

b)

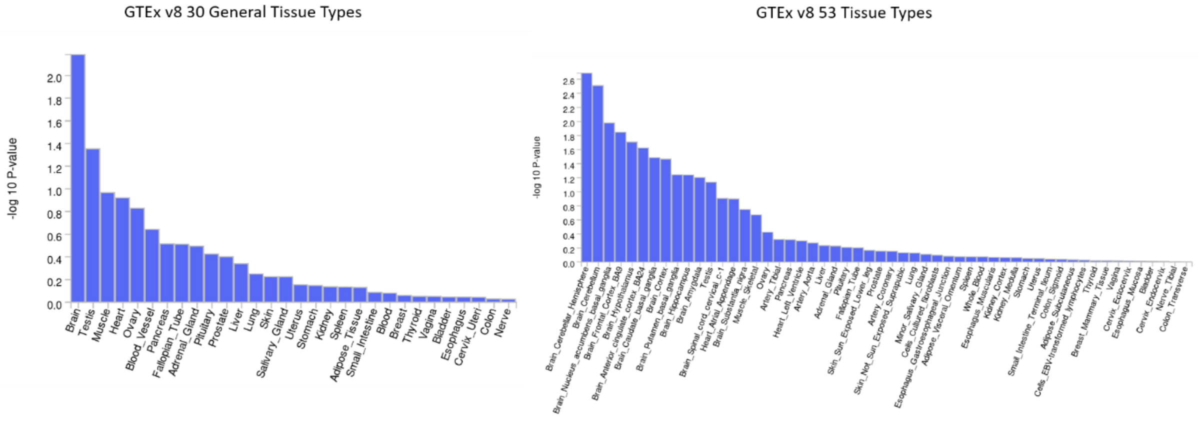

c)

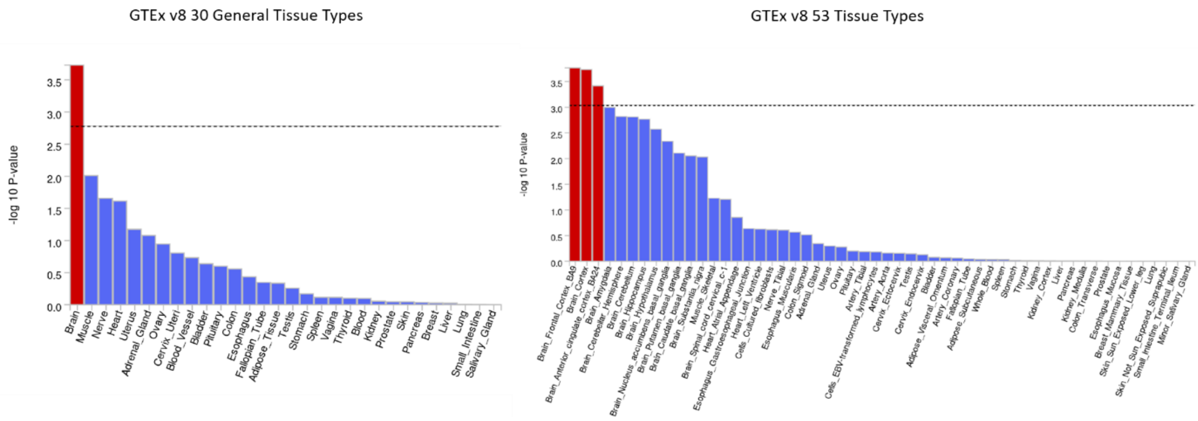
GTEx v8 30 General Tissue Types
-log 10 P-value
0.0
0.5
1.0
1.5
2.0
2.5
3.0
3.5
Brain
Muscle
Nerve
Heart
Uterus
Adrenal_Gland
Ovary
Cervix_Uteri
Blood_Vessel
Bladder
Pituitary
Colon
Esophagus
Fallopian_Tube
Adipose_Tissue
Testis
Stomach
Spleen
Vagina
Thyroid
Blood
Kidney
Prostate
Skin
Pancreas
Breast
Liver
Lung
Small_Intestine
Salivary_Gland
GTEx v8 53 Tissue Types
-log 10 P-value

d)

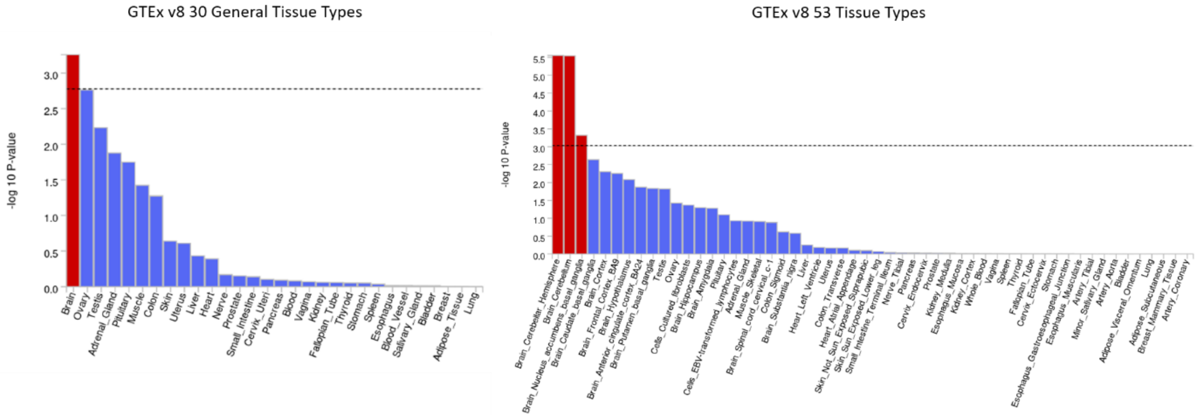
GTEx v8 30 General Tissue Types
-log 10 P-value
0.0
0.5
1.0
1.5
2.0
2.5
3.0
Brain
Ovary
Testis
Adrenal_Gland
Pituitary
Muscle
Colon
Skin
Uterus
Liver
Heart
Nerve
Prostate
Small_Intestine
Cervix_Uteri
Pancreas
Blood
Vagina
Kidney
Fallopian_Tube
Thyroid
Stomach
Spleen
Esophagus
Blood_Vessel
Salivary_Gland
Bladder
Breast
Adipose_Tissue
Lung
GTEx v8 53 Tissue Types
-log 10 P-value

Supplementary Figure 11. Circular plots of functional mapping for accelerometer-based sleep traits: a) night-time sleep duration; b) sleep efficiency; c) REM sleep; d) Non-REM sleep. Genomic risk loci are highlighted in blue. Genes mapped only by chromatin interactions in orange, only by eQTLs in green, by both in red. Links colored orange are chromatin interactions, links colored green are eQTLs.

a)

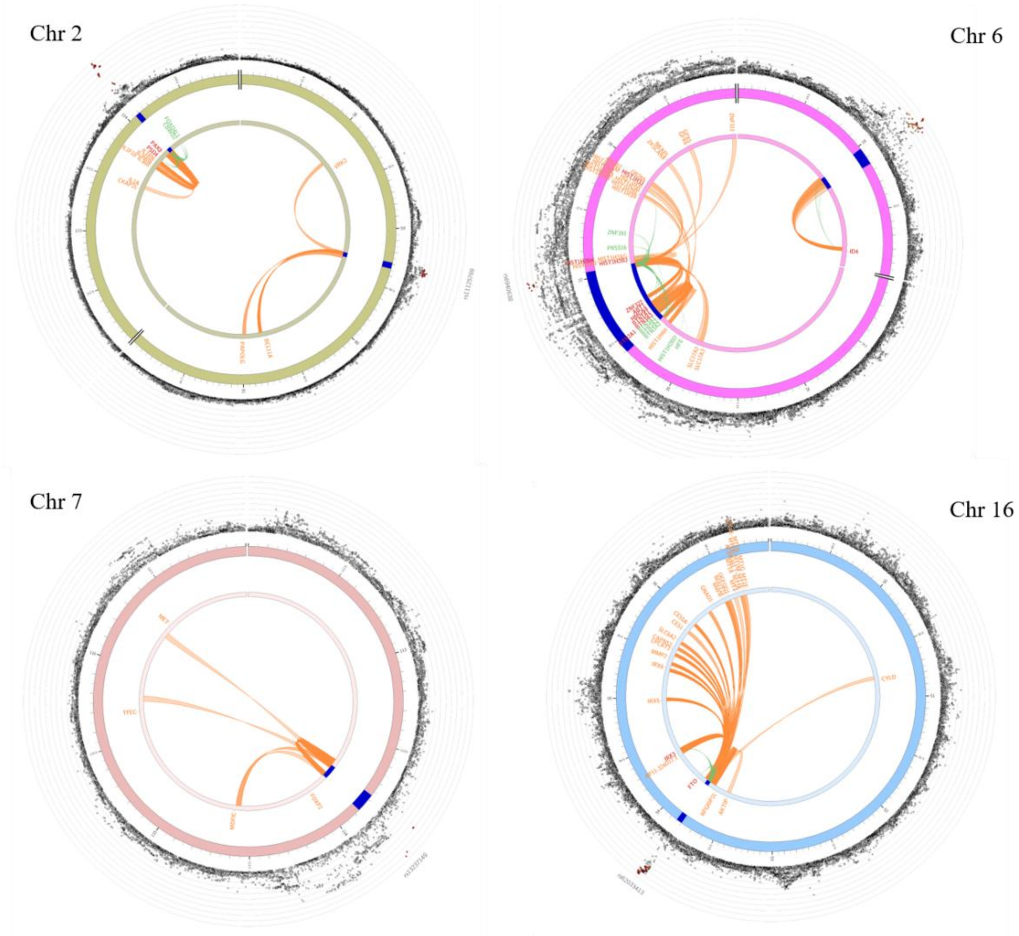

b)

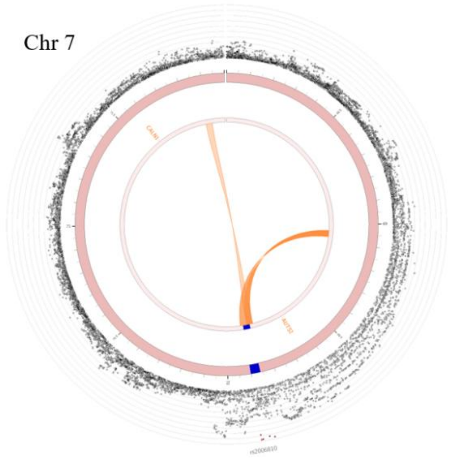

c)

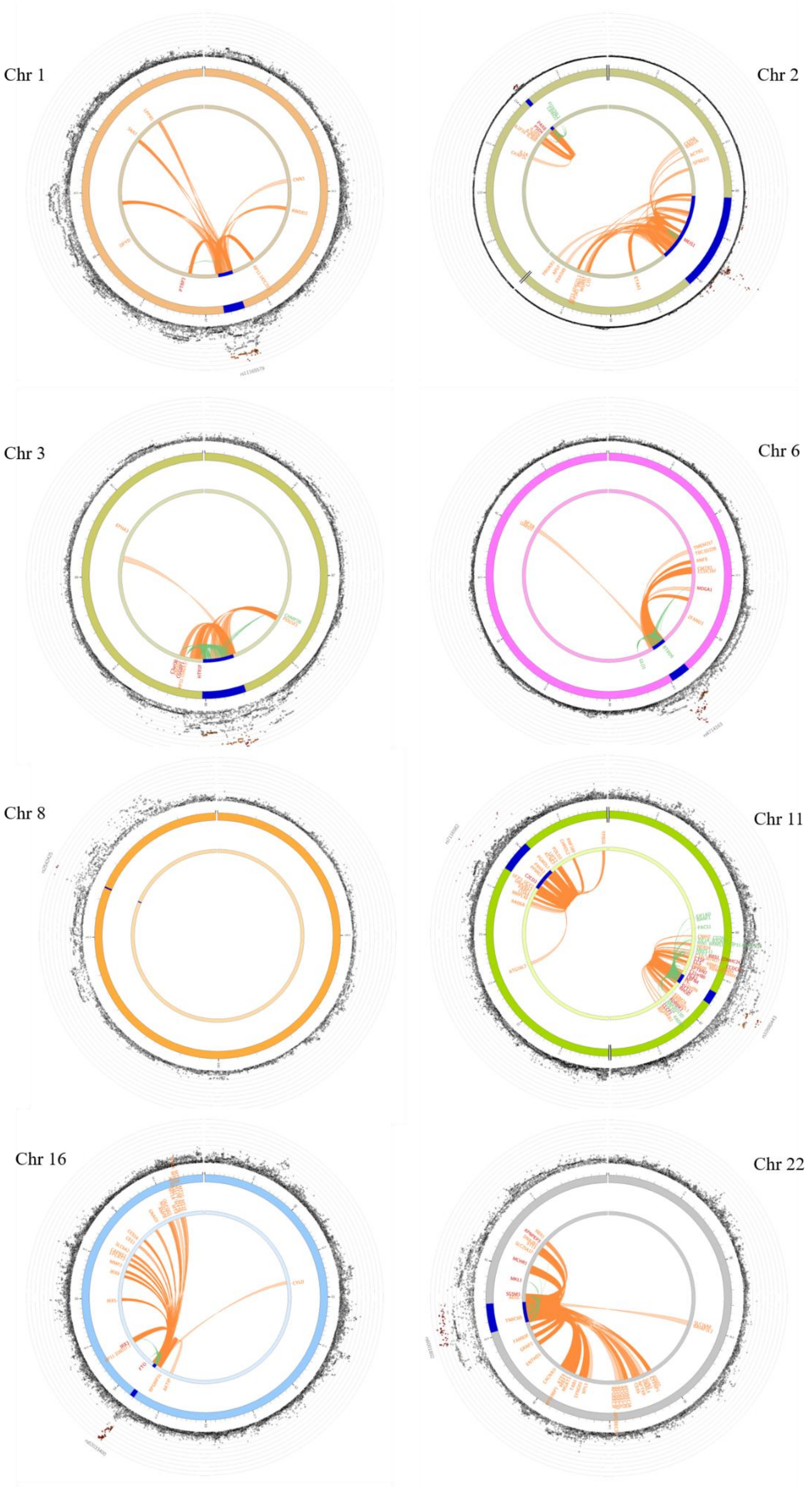

d)

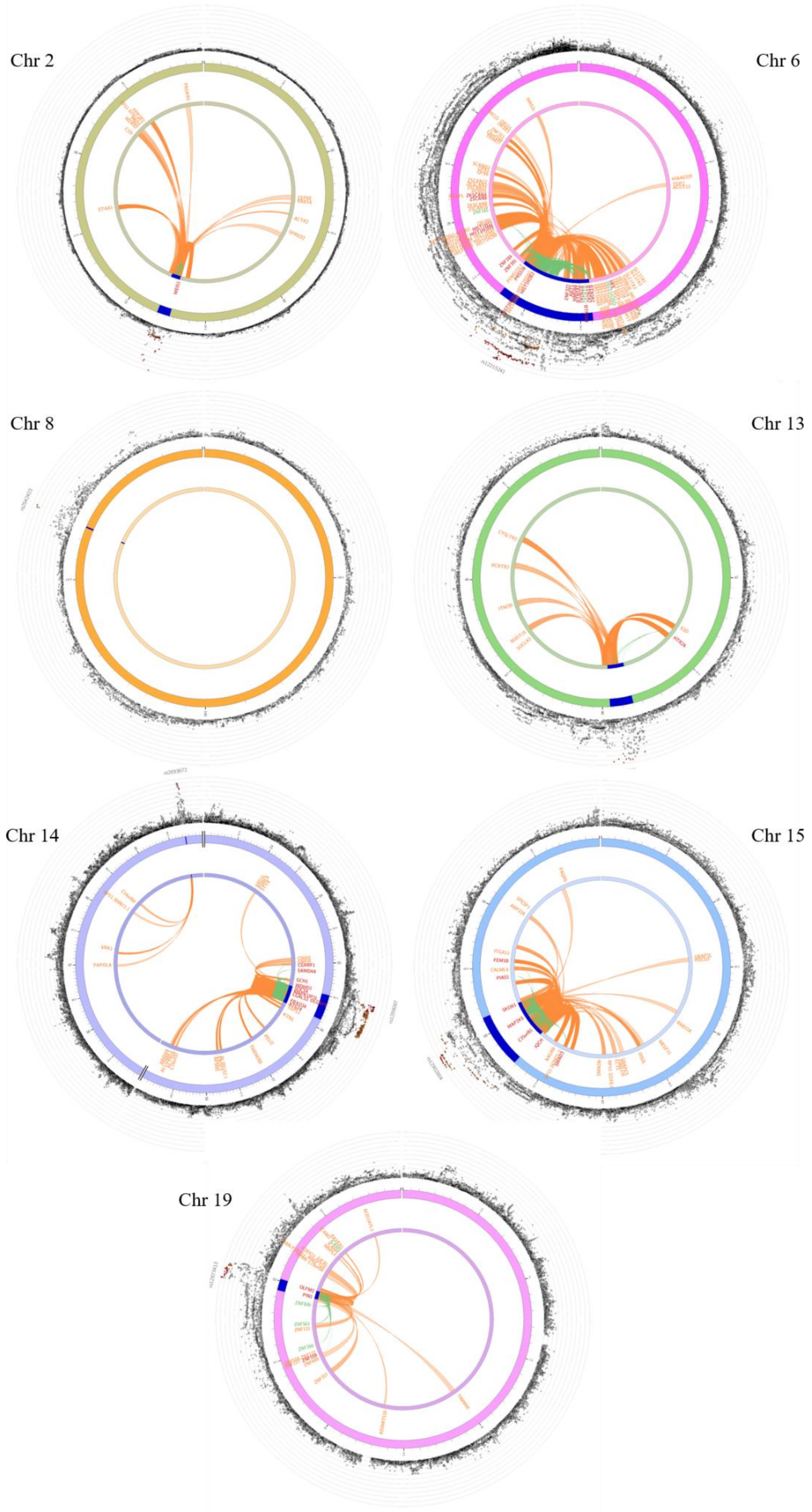

Supplementary Figure 12. LocusCompare plots of significant GWAS-eQTL colocalizations with a p-value ≤ 5 x 10^{-8} in both GWAS and eQTL data for sleep traits (night-time sleep: i) at the 2q14.1 locus for FOXD4L1 in the thyroid, ii) at the 2q14.1 locus for CBWD2 in the thyroid; REM sleep: iii) at the 2q14.1 locus for FOXD4L1 in the thyroid, iv) at the 2q14.1 locus for CBWD2 in the thyroid, v) at the 3p11.1 locus for HTR1F in the adipose visceral omentum, vi) at the 3p11.1 locus for HTR1F in the heart left ventricle, vii) at the 3p11.1 locus for HTR1F in the spleen, viii) at the 3p11.1 locus for HTR1F in the thyroid; Non-REM sleep: ix) at the 6p22.2 locus for BTN2A2 in the pancreas, x) at the 6p22.2 locus for HIST1H2BK in the tibial nerve, xi) at the 14q22.3 locus for WDHD1 in the esophagus mucosa, xii) at the 15q23 locus for SKOR1 in the thyroid, xiii) at the 19p13.2 locus for PIN1DT in the brain frontal cortex). Left hand panel represents GWAS (x-axis) and eQTL (y-axis) -log p values for the gene variants at the locus. Right hand panels represent association data with the -log p values on the y-axis for the eQTL (upper panel) and GWAS (lower panel).

i)

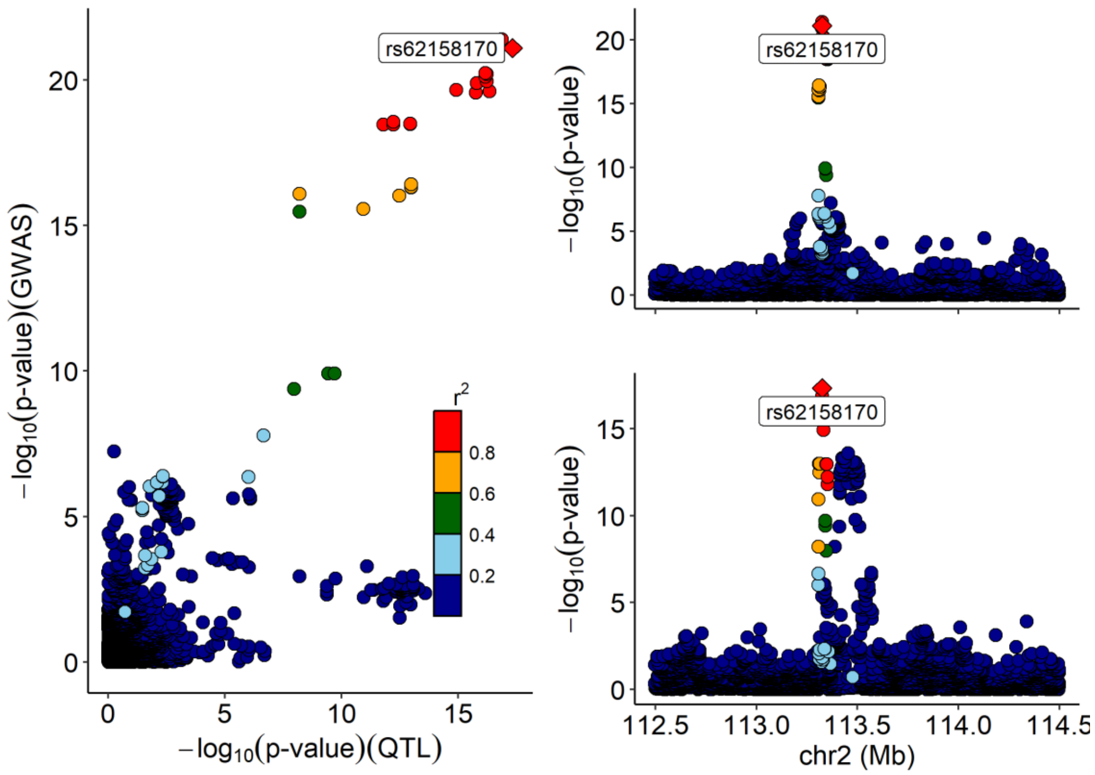

ii)

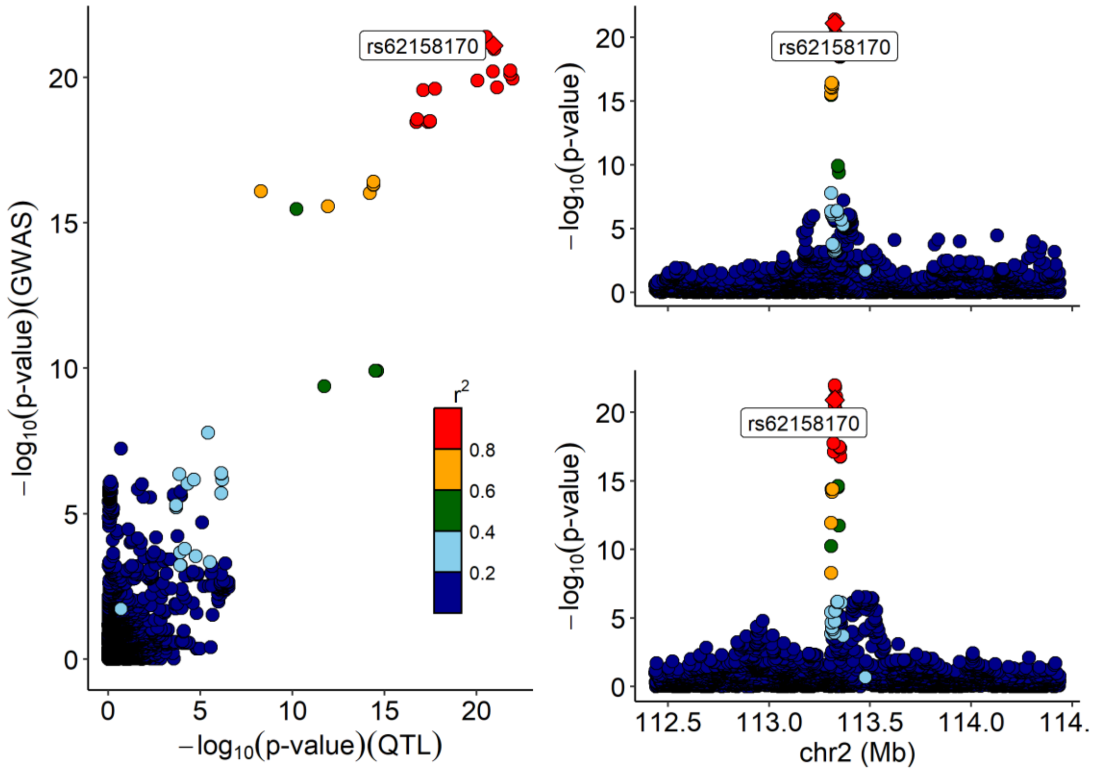
rs62158170
−log10(p-value)(GWAS)
−log10(p-value)(QTL)
r2
0.8
0.6
0.4
0.2
rs62158170
−log10(p-value)
rs62158170
−log10(p-value)
112.5 113.0 113.5 114.0
chr2 (Mb)

iii)

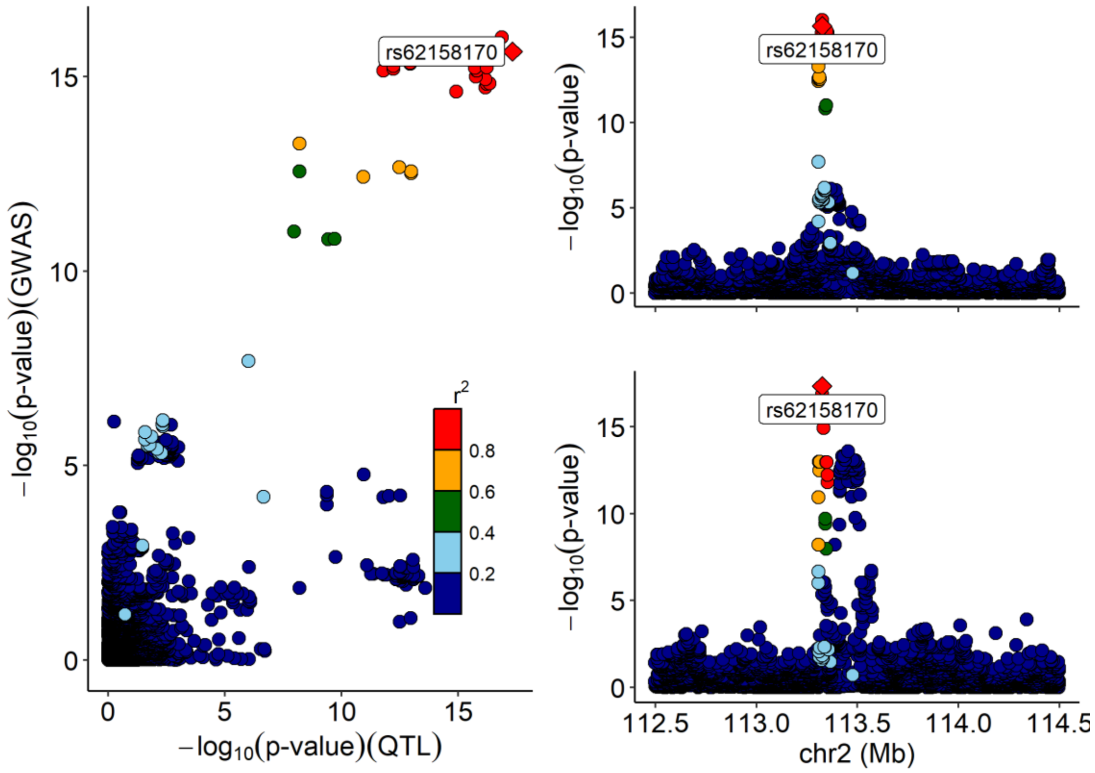
rs62158170
−log10(p-value)(GWAS)
−log10(p-value)(QTL)
r2
0.8
0.6
0.4
0.2
rs62158170
−log10(p-value)
rs62158170
−log10(p-value)
112.5 113.0 113.5 114.0 114.5
chr2 (Mb)

iv)

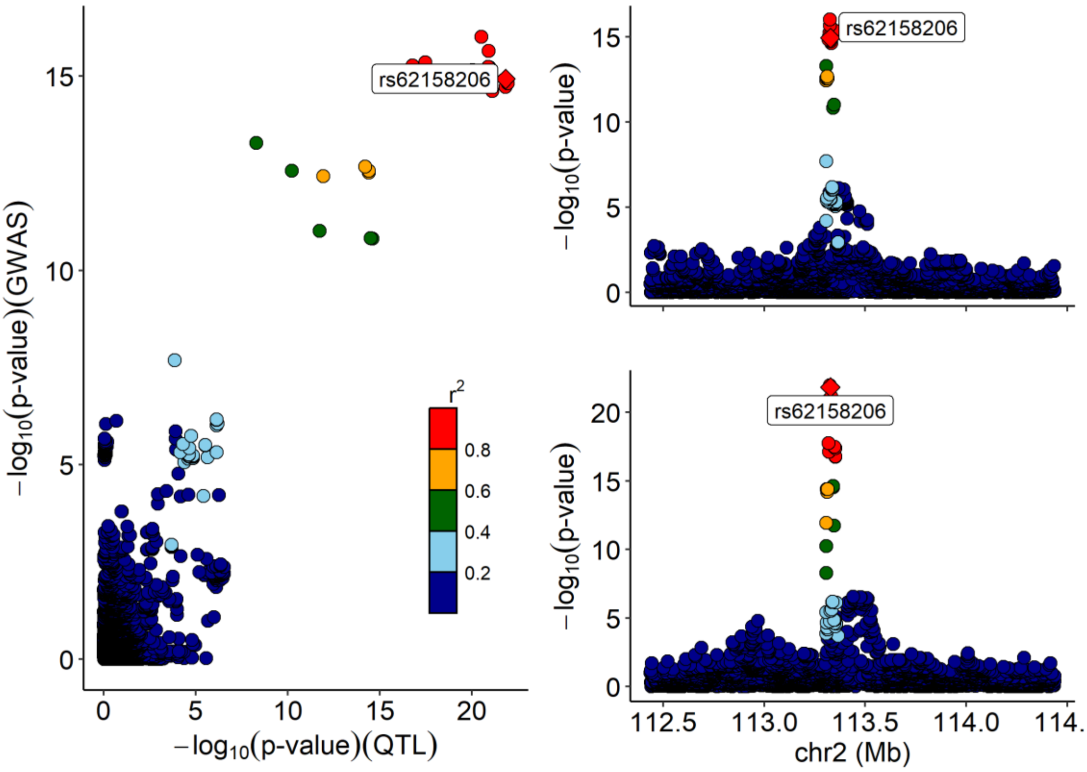
rs62158206
15
10
5
0
0
5
10
15
20
−log10(p-value)(QTL)
−log10(p-value)(GWAS)
r2
0.8
0.6
0.4
0.2
rs62158206
−log10(p-value)
rs62158206
20
112.5
113.0
113.5
114.0
114.
chr2 (Mb)

v)

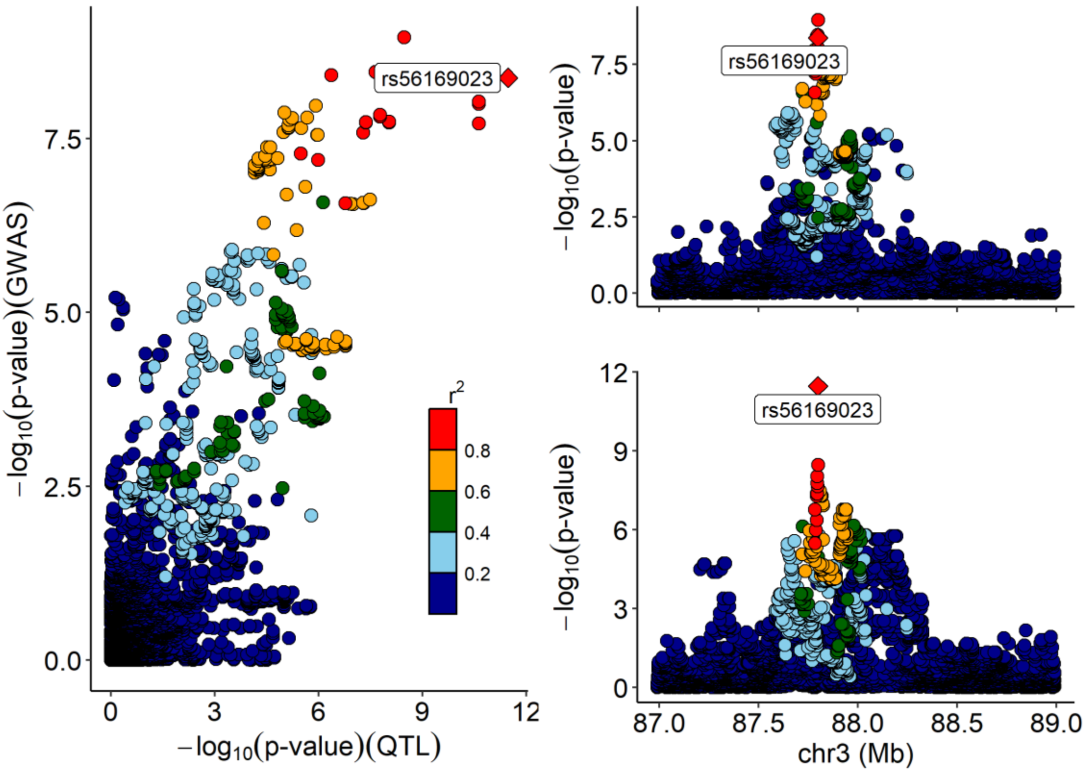
rs56169023
7.5
5.0
2.5
0.0
0
3
6
9
12
−log10(p-value)(QTL)
−log10(p-value)(GWAS)
r2
0.8
0.6
0.4
0.2
rs56169023
−log10(p-value)
rs56169023
87.0
87.5
88.0
88.5
89.0
chr3 (Mb)

vi)

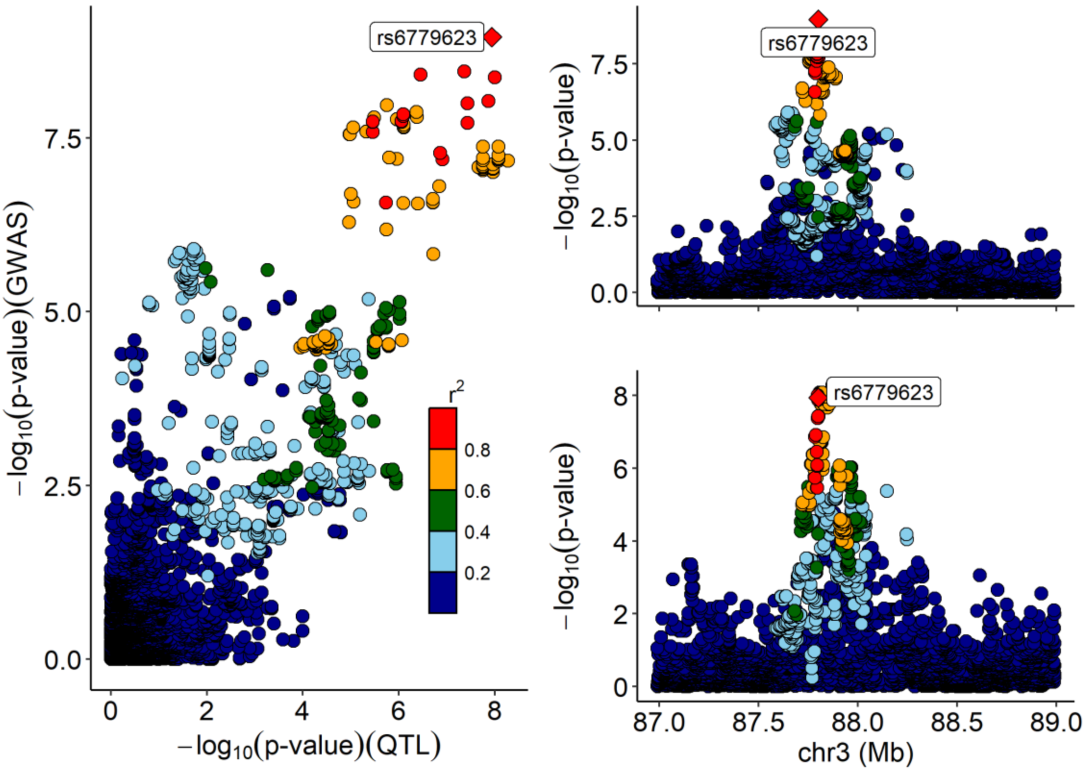

rs6779623
7.5
5.0
2.5
0.0
0
2
4
6
8
–log10(p-value)(GWAS)
−log10(p-value)(QTL)
r2
0.8
0.6
0.4
0.2
rs6779623
−log10(p-value)
rs6779623
8
6
4
2
0
87.0
87.5
88.0
88.5
89.0
chr3 (Mb)

vii)

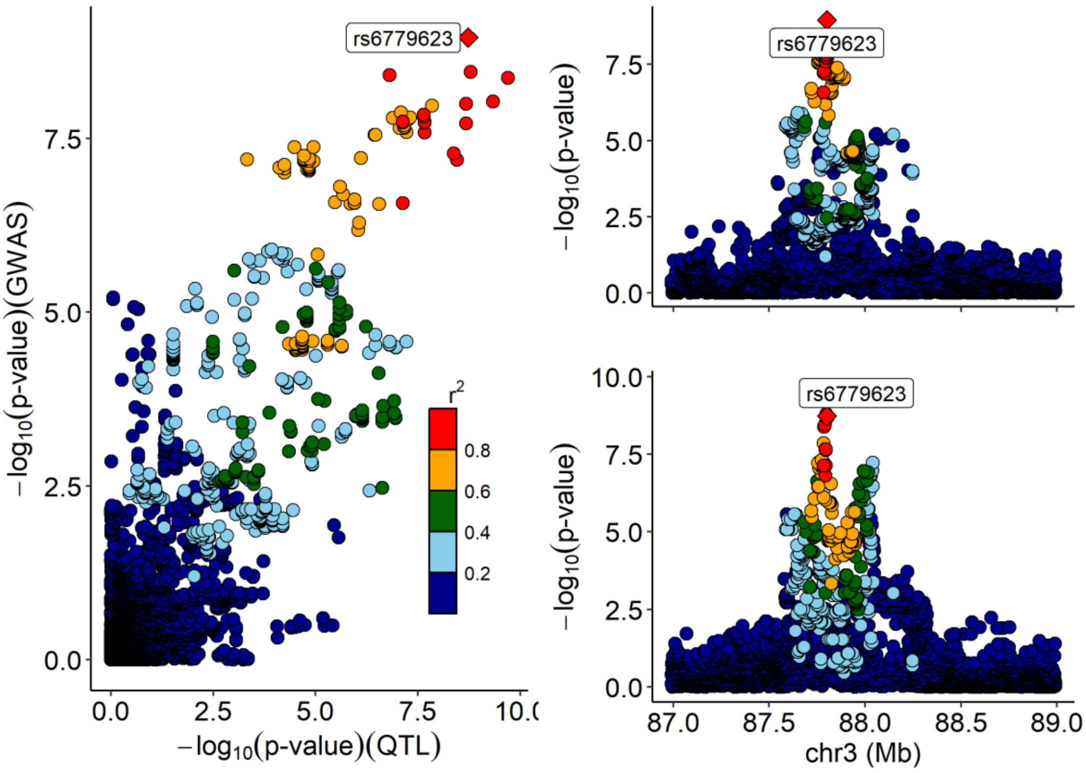

rs6779623
7.5
5.0
2.5
0.0
0.0
2.5
5.0
7.5
–log10(p-value)(GWAS)
−log10(p-value)(QTL)
r2
0.8
0.6
0.4
0.2
rs6779623
−log10(p-value)
rs6779623
10.0
87.0
87.5
88.0
88.5
89.0
chr3 (Mb)

viii)

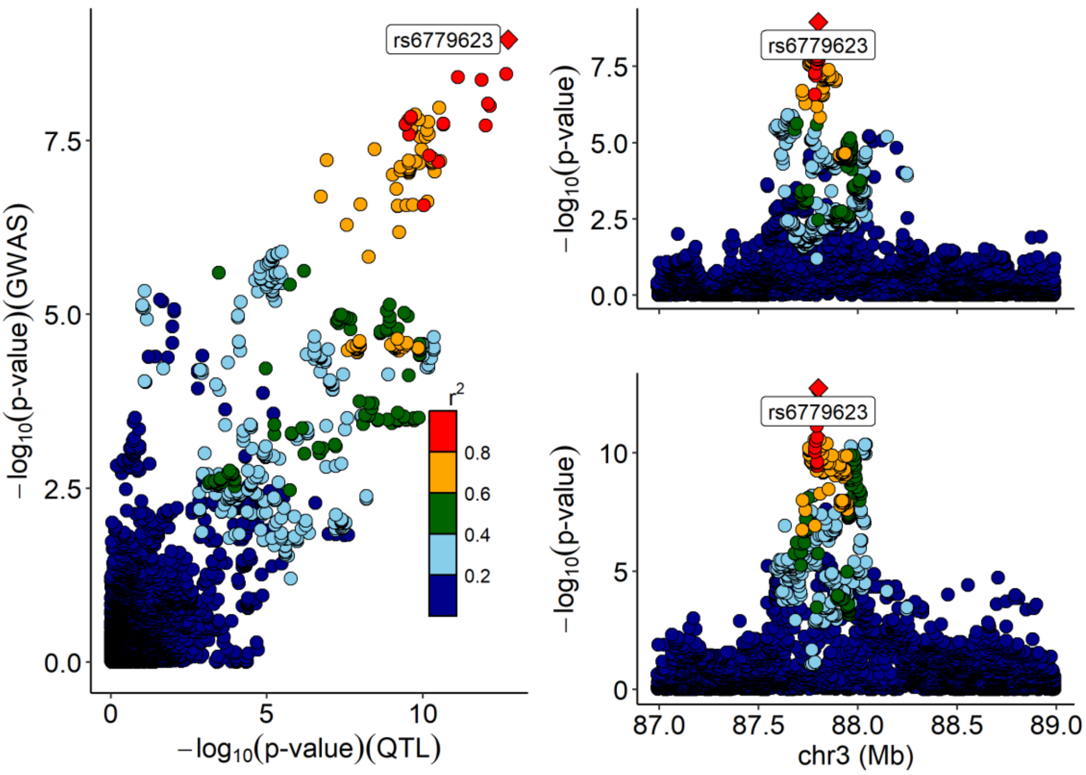
rs6779623
-log10(p-value)(GWAS)
-log10(p-value)(QTL)
r2
0.8
0.6
0.4
0.2
-log10(p-value)
chr3 (Mb)
87.0
87.5
88.0
88.5
89.0

ix)

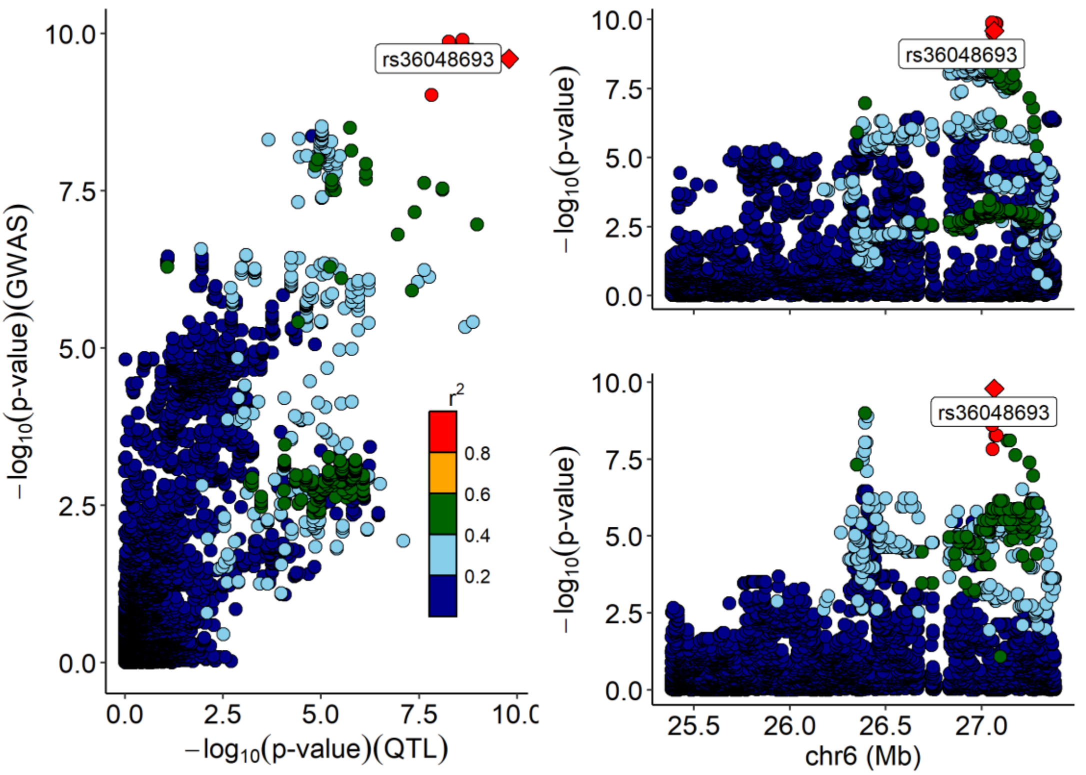
rs36048693
-log10(p-value)(GWAS)
-log10(p-value)(QTL)
r2
0.8
0.6
0.4
0.2
-log10(p-value)
chr6 (Mb)
25.5
26.0
26.5
27.0

x)

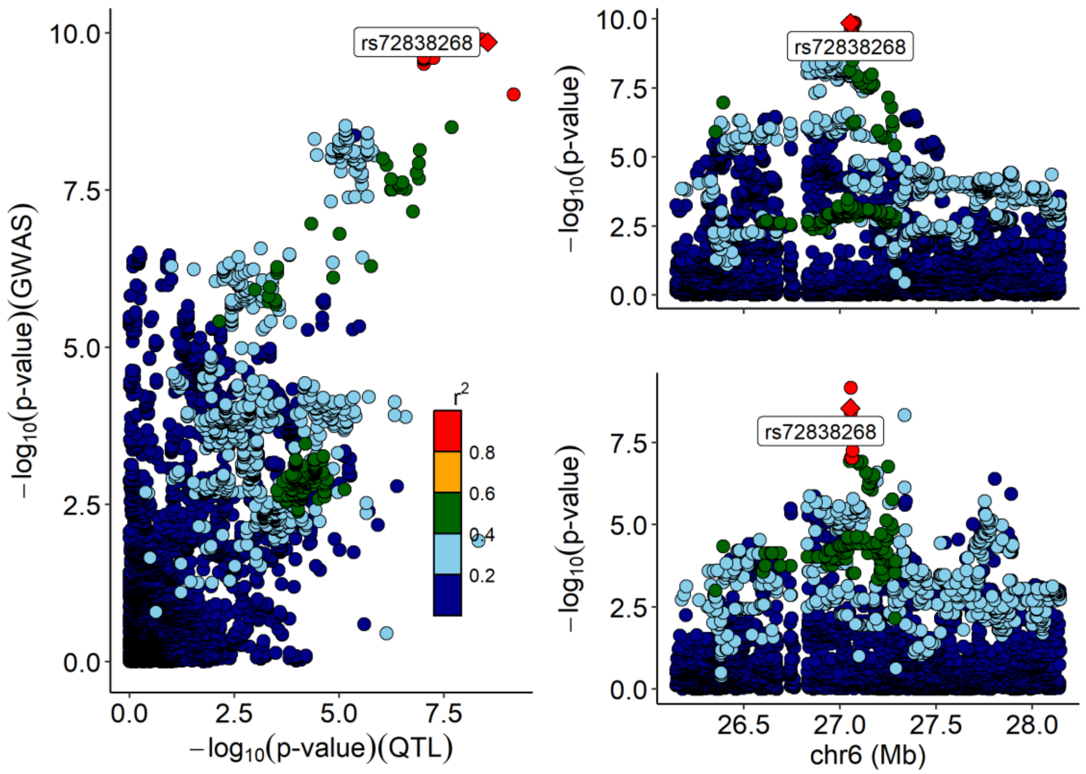
rs72838268
10.0
7.5
5.0
2.5
0.0
−log10(p-value)(GWAS)
0.0
2.5
5.0
7.5
−log10(p-value)(QTL)
r2
0.8
0.6
0.4
0.2
−log10(p-value)
26.5
27.0
27.5
28.0
chr6 (Mb)

xi)

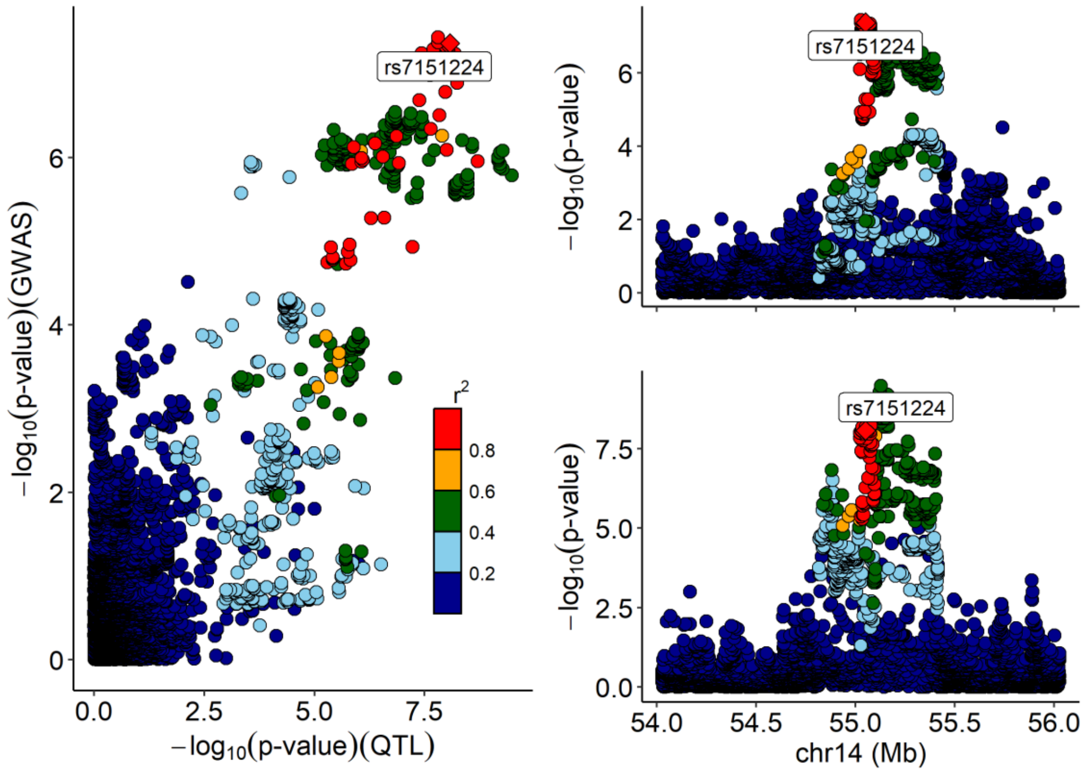
rs7151224
6
4
2
0
−log10(p-value)(GWAS)
0.0
2.5
5.0
7.5
−log10(p-value)(QTL)
r2
0.8
0.6
0.4
0.2
−log10(p-value)
54.0
54.5
55.0
55.5
56.0
chr14 (Mb)

xii)

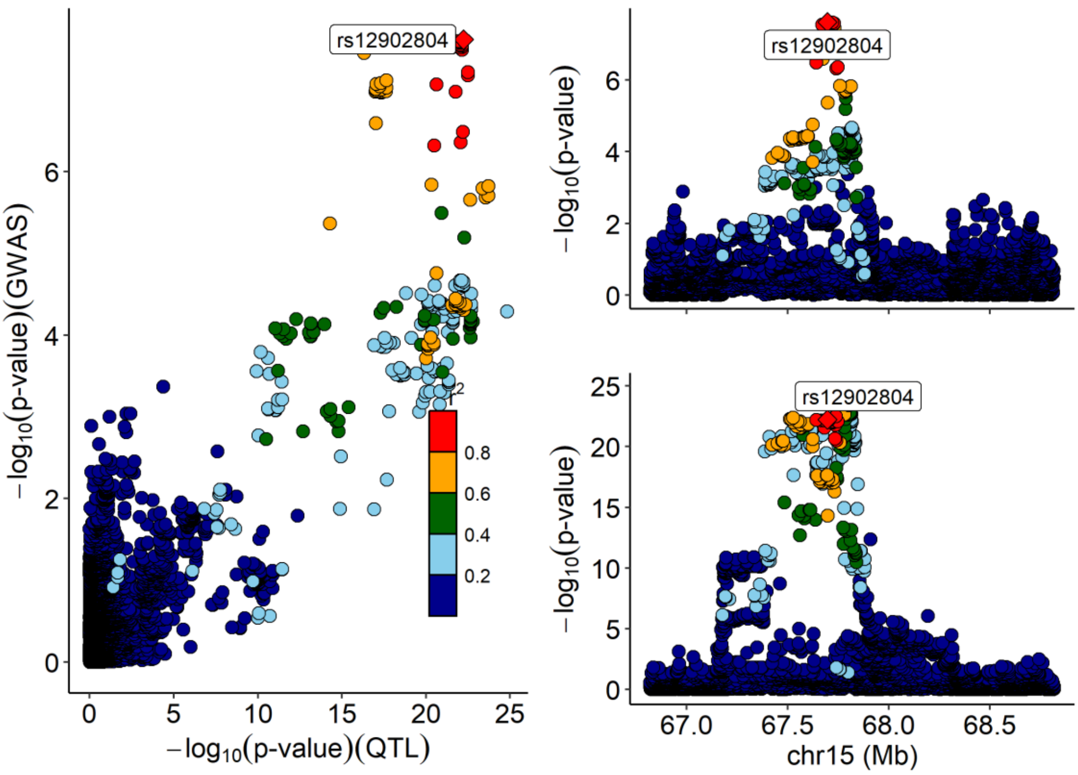
rs12902804
-log10(p-value)(GWAS)
-log10(p-value)(QTL)
r2
0.8
0.6
0.4
0.2
rs12902804
-log10(p-value)
rs12902804
-log10(p-value)
67.0
67.5
68.0
68.5
chr15 (Mb)

xiii)

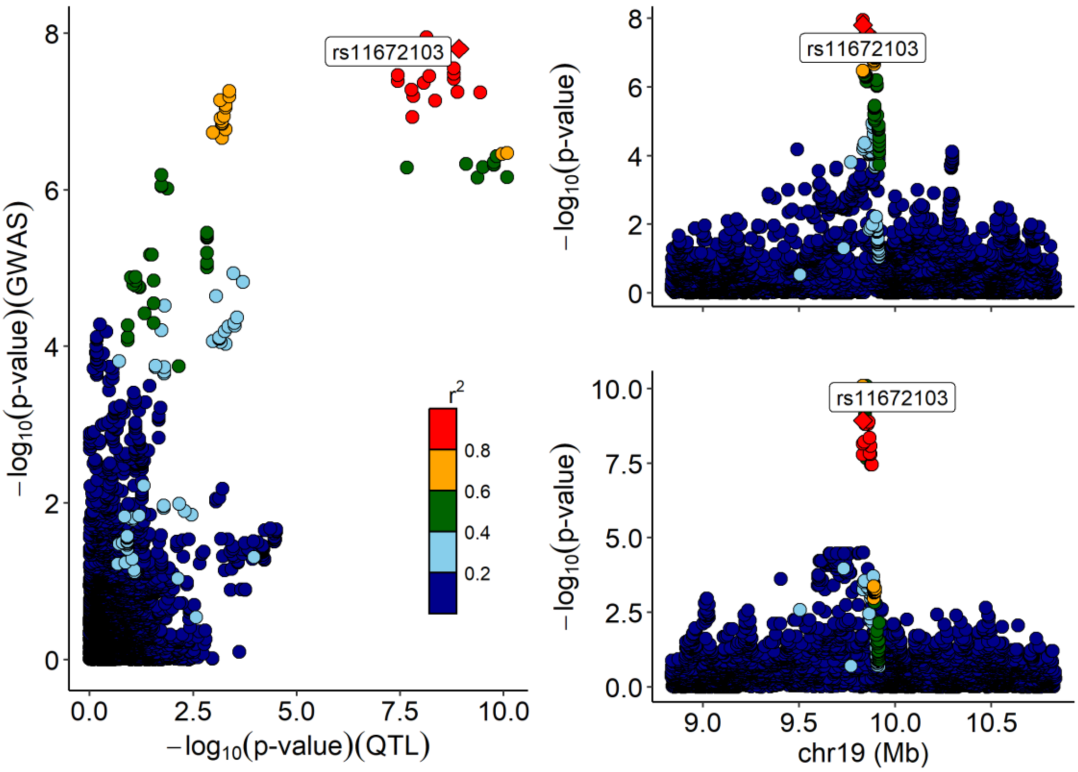
rs11672103
-log10(p-value)(GWAS)
-log10(p-value)(QTL)
r2
0.8
0.6
0.4
0.2
rs11672103
-log10(p-value)
rs11672103
-log10(p-value)
9.0
9.5
10.0
10.5
chr19 (Mb)

Supplementary Figure 13. Scatter plots of genetic associations with the outcome versus the exposure from bidirectional Mendelian randomization analyses meeting our significance or suggestive thresholds. Lines are shown for each applicable method (IVW, MR-Egger, weighted median, MR-RAPS). X-axis: SNP effect on the exposure; Y-axis: SNP effect on the outcome. Plots are provided only for analyses with ≥3 instruments.

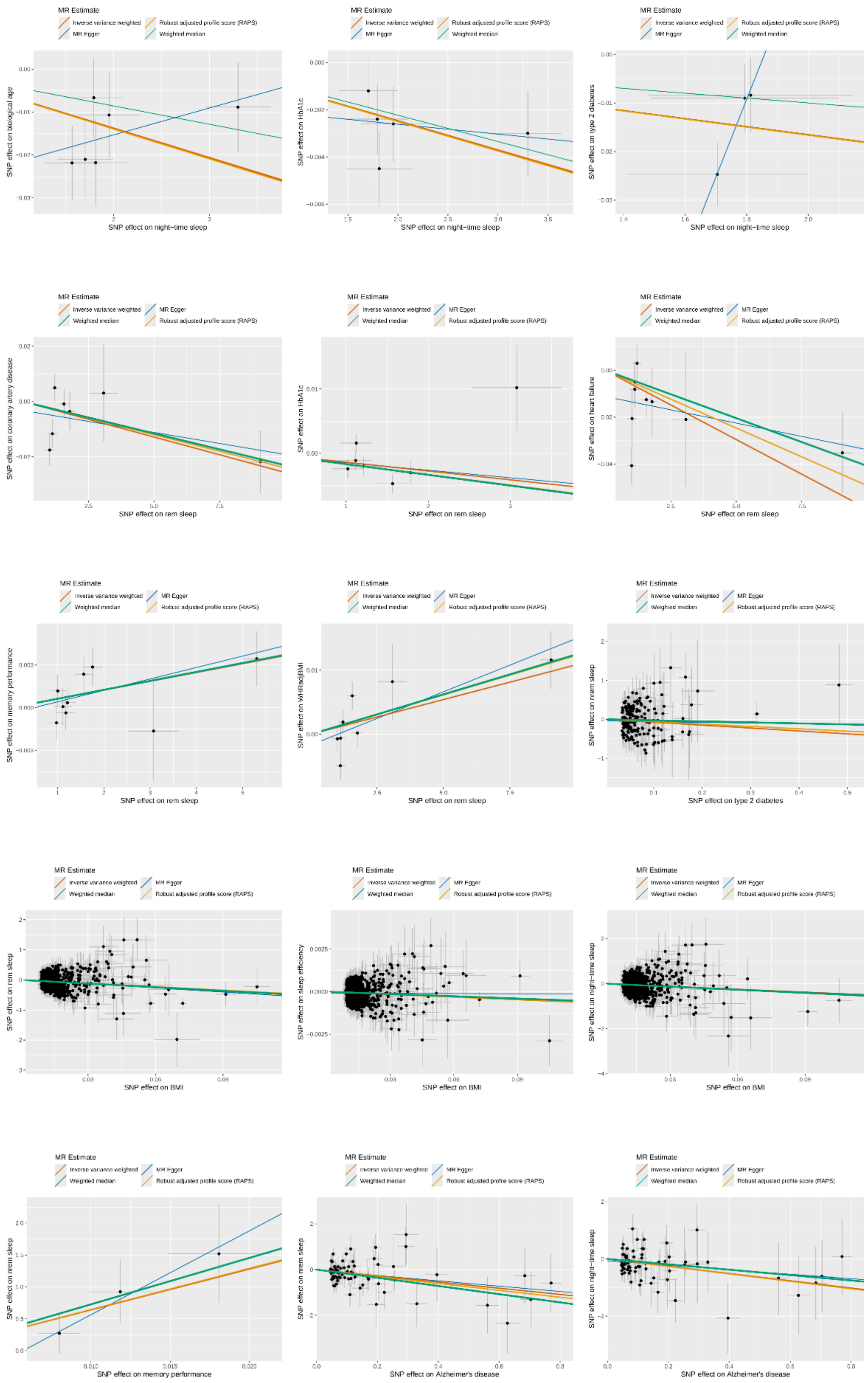

Supplement: Supplementary file 1 — Supplementary Information [file 41467_2026_71252_MOESM1_ESM.pdf]
