# Supplementary material for: Genetic architecture of sleep in a genome wide association study of device measured sleep traits
Source: Nat Commun. 2026 Apr 1;17:4715. doi: 10.1038/s41467-026-71252-y (PMC13212655; doi:10.1038/s41467-026-71252-y)
Supplement: Supplementary file 2 — Description of Additional Supplementary Files [file 41467_2026_71252_MOESM2_ESM.pdf]

Description of Additional supplementary files

Supplementary Data1: Participant characteristics in the overall sample and by sex.

Supplementary Data2: GWAS Catalog lookup of variants in linkage disequilibrium with genome-wide significant SNPs.

Supplementary Data3: Results of multitrait analysis of genome-wide association studies (MTAG).

Supplementary Data4: SuSiE fine-mapping results. Supplementary Data5: Sensitivity analyses for genome-wide significant loci. Supplementary Data6: Sex-stratified GWAS results.

Supplementary Data7: MAGMA gene-set enrichment analysis results.

Supplementary Data8: Exploratory cell-type enrichment analysis using FUMA.

Supplementary Data9: Gene-based association results obtained using MAGMA.

Supplementary Data10: Prioritized genes identified by functional mapping.

Supplementary Data11: GWAS–eQTL colocalization results (PP.H4 ≥ 75%).

Supplementary Data12: Brain-specific eQTL evidence for genes associated with sleep traits.

Supplementary Data13: Bidirectional two-sample Mendelian randomization results.

Supplementary Data14: Definitions considered for exclusions in the sensitivity analyses.

Supplementary Data15: List of genes tested in the GWAS-eQTL colocalization analysis.

Supplementary Data16: List of tissues tested in the GWAS-eQTL colocalization analysis.

Supplementary Data17: List of instrumental variables used in Mendelian randomization analyses.
